# Peptide–oligonucleotide conjugates as nanoscale building blocks for assembly of an artificial three-helix protein mimic

**DOI:** 10.1038/ncomms12294

**Published:** 2016-07-28

**Authors:** Chenguang Lou, Manuel C. Martos-Maldonado, Charlotte S. Madsen, Rasmus P. Thomsen, Søren Roi Midtgaard, Niels Johan Christensen, Jørgen Kjems, Peter W. Thulstrup, Jesper Wengel, Knud J. Jensen

**Affiliations:** 1 Department of Physics, Chemistry and Pharmacy, Biomolecular Nanoscale Engineering Center, University of Southern Denmark, Campusvej 55, Odense M 5230, Denmark; 2 Department of Chemistry, Biomolecular Nanoscale Engineering Center, University of Copenhagen, Thorvaldsensvej 40, Frederiksberg 1871, Denmark; 3 Biomolecular Nanoscale Engineering Center and Interdisciplinary Nanoscience Center (iNANO), University of Aarhus, Gustav Wieds Vej 14, Aarhus C 8000, Denmark; 4 Niels Bohr Institute, University of Copenhagen, Universitetsparken 5, Copenhagen Ø 2100, Denmark; 5 Department of Chemistry, University of Copenhagen, Universitetsparken 5, Copenhagen Ø 2100, Denmark

## Abstract

Peptide-based structures can be designed to yield artificial proteins with specific folding patterns and functions. Template-based assembly of peptide units is one design option, but the use of two orthogonal self-assembly principles, oligonucleotide triple helix and a coiled coil protein domain formation have never been realized for *de novo* protein design. Here, we show the applicability of peptide–oligonucleotide conjugates for self-assembly of higher-ordered protein-like structures. The resulting nano-assemblies were characterized by ultraviolet-melting, gel electrophoresis, circular dichroism (CD) spectroscopy, small-angle X-ray scattering and transmission electron microscopy. These studies revealed the formation of the desired triple helix and coiled coil domains at low concentrations, while a dimer of trimers was dominating at high concentration. CD spectroscopy showed an extraordinarily high degree of α-helicity for the peptide moieties in the assemblies. The results validate the use of orthogonal self-assembly principles as a paradigm for *de novo* protein design.

## Abstract (web summary)

Peptide and oligonucleotide systems are known to self-assemble both in nature and artificial systems. Here, the authors combine both forms of self-assembly through the synthesis of peptideoligonucleotide conjugates and show formation of a three-helix structure that dimerises at higher concentrations.

## Graphical abstract

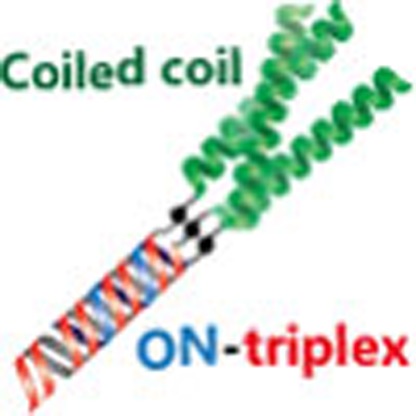

Designed peptide-based structures have been shown to yield artificial proteins^1^ and even in a few cases nanoscale objects^2^,^3^,^4^. Simultaneously, oligonucleotides (ONs) have been intensively used for nanotechnology^5^,^6^, including so-called DNA origami^7^, to create structurally advanced objects such as DNA-based nanoboxes^8^. In contrast, formation of nanoscale objects and artificial proteins from peptide-ON conjugates (POCs) as building blocks with combined involvement of two separate self-assembly principles have, to our knowledge, not yet been realized. Protein *de novo* design involves rational design of peptide or protein molecules to fold into a target protein or protein-like structure, rather than the use or re-design of a naturally occurring sequence. Protein *de novo* design offers a test of our understanding of the factors controlling protein structure, folding and stability. The approach also offers the prospect of access to tailor-made proteins^9^,^10^,^11^,^12^,^13^,^14^,^15^,^16^,^17^. One way to overcome the complexity of protein folding is the concept of template-assembled synthetic proteins (TASPs)^18^,^19^. Several groups have explored this approach with a diverse set of templates^20^,^21^,^22^,^23^,^24^,^25^, and we have reported the first low-resolution structure of a TASP in the form of a carboprotein, using small-angle X-ray scattering (SAXS)^26^.

Well-defined DNA secondary structures such as double helices^27^, triple helices^28^, multi-way junctions^29^ and quadruplexes^30^ are potential scaffolds in designs of TASPs, requiring POCs as building blocks. Different methods have previously been applied to couple peptides or proteins with ONs^31^, including azide-alkyne cycloaddition reactions due to their remarkable compatibility with diverse functional groups and their high second-order rate constant under mild conditions^32^,^33^. However, to the best of our knowledge, only three examples have been reported on peptide and protein assembly driven by the formation of DNA secondary structures^34^,^35^,^36^. In one report it was demonstrated that quadruplex formation could orient two very short peptides to form an adjacent two-loop protein-like surface^34^, in another that three- and four-way DNA junctions positioned one recombinant eADF4(C16) element at each terminal^35^, and in a more recent report that two model substrates (a maltose-binding protein and an antibody fragment against urokinase plasminogen activator receptor) positioned on DNA junctions imitated the geometry of an antibody^36^. Previously, POCs have thus not been explored in *de novo* protein design using two orthogonal self-assembly principles.

For the present proof-of-concept study we relied on a sequence derived from CoilV_a_L_d_^37^,^38^ which was N-terminally extended with an azidohexanoyl-Tyr linker. The α-helical coiled coil is a ubiquitous protein motif that exists in 5–10% of all protein sequences^39^. One of the main characteristics of a coiled coil is the simplicity of its sequence, as it consists of a motif that repeats itself every seven residues, (abcdefg)_n_. In coiled coils, two or more helices wrap around each other in a left- or right-handed helical twist conformation. These helices can adopt different topologies, as they can assemble in parallel or antiparallel orientation. The solution structure analysis of CoilV_a_L_d_ also revealed a cooperative monomer–dimer–trimer equilibrium, with the dimer state being an intermediate^37^. Later the crystal structure of CoilV_a_L_d_ revealed a parallel triple-helical structure^38^.

Herein, we report an efficient and high-yielding preparation of POCs by copper-free alkyne-azide cycloaddition reactions, and we show that ON triple helix-formation can be used to organize peptide strands leading to the formation of a highly stable three-helix bundle protein mimic that dimerizes at higher concentrations. Locked nucleic acid (LNA) was central to our design^40^.

## Results

### Synthesis of POCs

**Figure 1:**
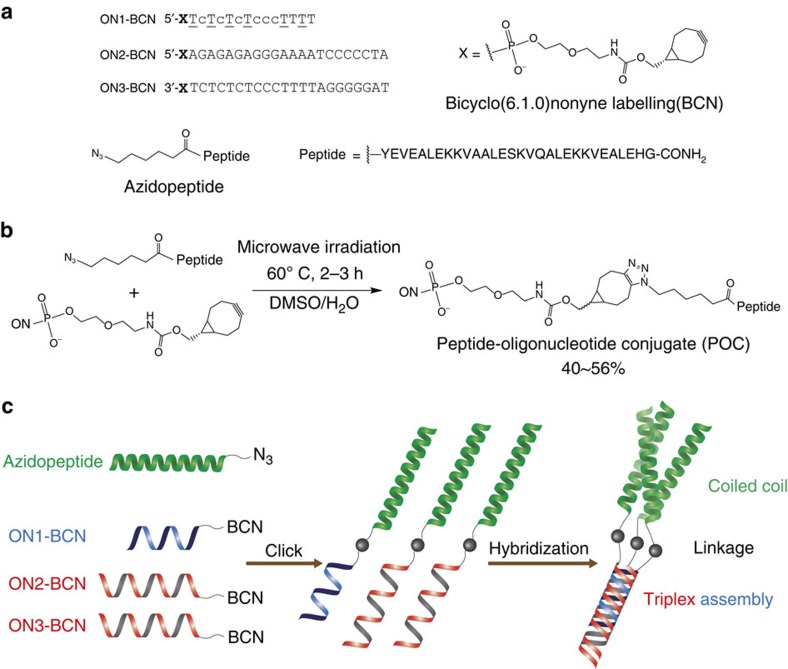
Design and assembly of the desired three-helix protein mimic. (**a**) Sequence of the azidopeptide and ON1-BCN, ON2-BCN and ON3-BCN: T=LNA-T and c=5-methyl-2'-deoxycytidine. A, C, G and T are DNA monomers. (**b**) Illustration of ring-strain promoted azide-alkyne cycloaddition chemistry between the azidopeptide and a BCN-containing ON. (**c**) Schematic illustration of the synthesis of POCs through azide-alkyne chemistry and formation of a triple-helical protein mimic.

A convenient and efficient method using copper-free ring-strain promoted azide-alkyne coupling reactions was developed for synthesis of POCs (Fig. 1a). Among strained cyclooctynes^41^,^42^, bicyclo[6.1.0]nonyne (BCN) was chosen due to its high second-order rate constant and commercial availability as a phosphoramidite monomer ready for use on an automated DNA synthesizer (Fig. 1b)^43^,^44^. In this way the ON building blocks ON1-BCN, ON2-BCN and ON3-BCN were obtained. The azido-labelled peptide was synthesized by automated Fmoc chemistry and N-terminal attachment of a 6-azido hexanoyl group. Efficient and high-yielding preparation of POCs containing long peptide fragments (≥30-mers) or proteins is challenging and only very few successful examples have been published via chemical synthesis^35^,^36^,^45^, but this method furnished the desired conjugates POC1, POC2 and POC3 in satisfactory yields for milligram-scale preparation (further details are included in Supplementary Discussion, Supplementary Methods and Supplementary Figs 1–6).

### Ultraviolet-melting studies and gel electrophoresis analysis

As a first step in studying the applicability of nucleic acid self-assembly towards organizing peptide bundles (Fig. 1c), the stability of duplexes and triple helices involving one or more POC(s) was evaluated (see also details in Supplementary Methods), with their ON-based counterparts as references (Table 1a). One aspect was to study if the helical structures were stabilized by a synergistic effect involving both the ON and the peptide moieties. A wavelength of 275 nm was used for ultraviolet thermal denaturation (‘melting') studies where the ultraviolet absorption difference between the triple helix and the underlying duplex is maximal (Supplementary Fig. 7). An ultraviolet-scan showed that the peptide unit did not display significant absorption at this wavelength (Supplementary Fig. 8). For interpretation of these studies it is assumed that any higher-order structures possibly formed (see sections below on gel electrophoretic, SAXS and TEM analysis) have no significant effect on annealing or denaturation of duplexes or triple helices.

In the case of duplex systems (Table 1b), the POC2+POC3 assembly (Table 1b, entry 4) showed substantially higher thermal stability than the ON2+ON3 duplex (Table 1b, entry 1), which we attribute to an additional interaction between the peptide moieties acting cooperatively with the Watson–Crick interactions of the ON units (Table 1b, entry 4). The melting curve of the POC2+POC3 duplex is well superimposed on the corresponding annealing curve (Supplementary Fig. 9), indicating that the peptide modification does not perturb the thermal reversibility of the duplex structure. Importantly, no stabilization was seen for assemblies containing only one peptide unit (Table 1b, entries 2 and 3), strongly supporting that interactions between the peptide units play an indispensable role in stabilizing the POC2+POC3 structure.

Eight triple-helical complexes were formed using ON1 and POC1 as triple-helix-forming ON (TFO) designed to bind in the major groove of the four duplexes (Table 1b, entries 5–12). A significant increase in melting temperature was observed for the POC1+POC2+POC3 triple helix (Table 1b, entry 6) when compared with the corresponding ON1+ON2+ON3 triple helix (Table 1b, entry 5). We ascribe this increased stability to the formation of a three-helix parallel peptide bundle, which for the non-conjugated peptides in solution was reported as the predominant structure^37^,^46^. Accordingly, the annealing curve for POC1+POC2+POC3 gave a clear triple-helix annealing transition, whereas that transition was not detected for the ON1+ON2+ON3 triple helix (Supplementary Fig. 10). As expected, there was no or only little improvement of triple-helix stability when ON1 was bound to the three duplexes containing one or two peptide units (Table 1b, entries 7–9, Supplementary Fig. 11). The remaining assemblies displayed in Table 1b (entries 10–12) involve POC1, where increased triple-helix stability was also observed when only one strand of the duplex contained a peptide moiety (Table 1b, entries 10 and 11, Supplementary Fig. 12). This observation again strongly indicates cooperative effects between nucleic acid hybridization and peptide assembly. This was further evidenced by the lack of triple-helix annealing transitions for the assemblies with only one peptide unit (Table 1b, entries 7, 8 and 12) or with the two peptide units on the two duplex-forming strands (Table 1b, entry 9).

For POC1+POC2+ON3 and POC1+ON2+POC3 where the triple-helix transitions were overlapping with duplex transitions (Table 1b, entries 10 and 11), the experiments were repeated at pH 7.5. The stability of parallel nucleic acid triple helices is pH dependent, and in near physiological conditions the triple helices become less stable when the pH increases, mainly due to de-protonation of N3 of cytosine nucleobases^47^. Under these conditions, both hybrid triple helices exhibited distinctive triple-helix melting and annealing transitions, whereas the transition temperatures for the underlying duplexes were unchanged (Supplementary Fig. 13).

Non-denaturing polyacrylamide gel electrophoresis (see Supplementary Methods for details) was used to study the assemblies discussed above (Table 1). Relatively low-voltage and low-temperature (4 °C) conditions minimized the possible dissociation of any triple helix during electrophoresis.

**Figure 2:**
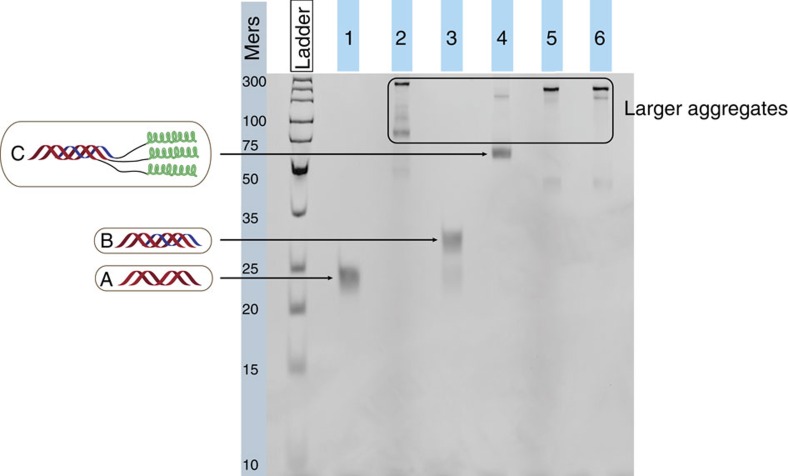
Gel analysis. Non-denaturing 13% PAGE at pH 7.0 and 4 °C: lane 1, ON2+ON3 (structure shown as ‘A'); lane 2, POC2+POC3; lane 3, ON1+ON2+ON3 (structure shown as ‘B'); lane 4, POC1+POC2+POC3 (structure shown as ‘C'); lane 5, ON2+POC3; lane 6, POC2+ON3. The gel was visualized by ultraviolet excitation at 260 nm after ethidium bromide staining. The O'GeneRuler Ultra Low Range DNA Ladder from bottom to top: 10, 15, 20, 25, 35, 50 75, 100, 150, 200 and 300 mers.

The reference ON2+ON3 assembly (Fig. 2, lane 1) appeared as a single band, consistent with the length of the duplex (22-mers). The ON1+ON2+ON3 assembly (Fig. 2, lane 3) appeared as one dominant band (triple helix), and a faint band corresponding to the ON2+ON3 duplex as a result of TFO dissociation (for room temperature conditions see Supplementary Fig. 14). When the pH was increased to 8.5 (Supplementary Figs 15 and 16) only the lower band was observed, consistent with the melting studies where no triple-helix transition was observed at pH 7.5 (Supplementary Fig. 13). The POC1+POC2+POC3 assembly (Fig. 2, lane 4) appeared as a dominating band with a far slower mobility than for the corresponding ON-based triple-helix control, confirming an increase in molecular size and mass-to-charge ratio due to the peptide moieties. In addition, a faint band moving close to the 150-mer DNA mark was observed which could be explained by dimerization (or multimerization) of the POC1+POC2+POC3 triple helix, possibly induced by additional intermolecular interactions. Similar behaviour was observed at three different conditions (pH 7.0, room temperature in Supplementary Fig. 14; pH 8.5, 4 °C in Supplementary Fig. 15; and pH 8.5, room temperature in Supplementary Fig. 16), demonstrating the existence of POC1+POC2+POC3 as a triple helix even at pH 8.5 at room temperature (Supplementary Fig. 16, lane 4) and the remarkable peptide-induced stabilization of the otherwise rather unstable ON triple helix. In contrary to the POC1+POC2+POC3 assembly, the POC2+POC3 as well as other bimolecular assemblies appeared as several bands with a lower mobility than the band for the POC1+POC2+POC3 triple helix (Fig. 2, lanes 2, 5 and 6) probably reflecting the formation of various multimeric higher-order structures induced by a drive towards formation of three-stranded peptide domains.

### Circular dichroism spectroscopy

**Figure 3:**
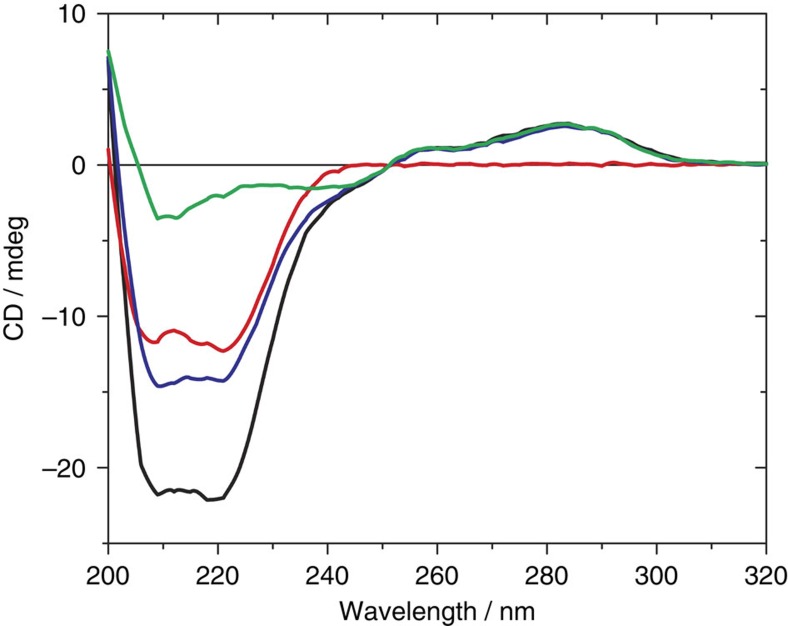
CD analysis. Far-ultraviolet CD spectra of POC1+POC2+POC3 (3:2:2 μM; black), azidopeptide (7μM; red), ON1+ON2+ON3 (3:2:2 μM; green) and a control containing azidopeptide (7 μM) and ON1+ON2+ON3 (3:2:2 μM; blue) at 20 °C in 5.8 mM phosphate buffer pH 7.0 with 100 mM NaCl and 0.10 mM EDTA. The spectra were recorded using a 0.2 cm path-length cell. The corresponding reference (buffer solution) was subtracted from each spectrum but no other manipulation was applied.

Figure 3 shows the circular dichroism (CD) spectra of POC1+POC2+POC3, azidopeptide, ON1+ON2+ON3 and a sample containing azidopeptide and triple-helix ON1+ON2+ON3 at 20 °C (see also Supplementary Methods for details). As expected, the far-ultraviolet CD spectrum of azidopeptide exhibited an intense α-helix signal with negative peaks at 222 and 208 nm, respectively. ON1+ON2+ON3 showed a wide positive signal between 305 and 251 nm with a maximum at 284 nm and a shoulder around 260 nm, a wide negative signal between 251 and 206 nm with a shoulder around 240 nm and a maximum around 211 nm, which corresponds to the spectrum of a hybridized B-form DNA^48^. Similarly, the spectral features in the high-wavelength region also indicated the formation of an ON triple helix (Supplementary Fig. 18). In Fig. 3, the higher wavelengths of the spectrum of POC1+POC2+POC3 were characterized by the contribution of the triple-helix ON signals with a wide positive feature between 305 and 251 nm with a maximum around 284 nm. At lower wavelengths, an intense negative signal between 251 and 201 nm was attributed to the peptide α-helix element with only a minor contribution by the ON region.

The potential intermolecular interaction between non-conjugated peptides and ONs were tested in a control experiment, where it was found that the spectrum of azidopeptide and ON1+ON2+ON3 matches the mathematical sum of the spectra of its separated components (Supplementary Fig. 19). Thus, conformational changes induced by interactions between unconjugated peptides and ONs can be excluded.

Importantly, the spectrum of POC1+POC2+POC3 was significantly different from the sum of the azidopeptide and ON1+ON2+ON3 spectra, but only in the far-ultraviolet region where the peptide transitions dominate (Fig. 3). In the near-ultraviolet region the positive signals observed for the ON samples between 305 and 251 nm were practically identical to those of the POC1+POC2+POC3 sample. The latter, however, exhibited a significantly more intense α-helix signal, which indicated a higher degree of α-helicity. The θ_222 nm_/θ_208 nm_ ratios, assumed to be ≥1 for coiled coil peptides, were calculated^49^,^50^. Azidopeptide and POC1+POC2+POC3 (after subtracting the spectrum of ON1+ON2+ON3 to remove the contribution of the ON part) showed θ_222 nm_/θ_208 nm_ ratios equal to 1.03 and 1.05, respectively. These values are consistent with the existence of stable α-helical coiled coils in solution and POC1+POC2+POC3 having a markedly increased helical content (see discussion below).

The concentration dependence of the helical content of POC1+POC2+POC3 and azidopeptide were tested by CD spectroscopy (Supplementary Discussion). Compared with the dilute samples shown in Fig. 3, a further change in the mean residue ellipticity was observed when the concentration was increased, both for azidopeptide and POC1+POC2+POC3. However, the latter exhibited a markedly increased CD signal magnitude in the concentration range tested. For the azidopeptide a plateau was reached at ∼15 μM with mean residue ellipticity values at 222 nm around −31,000 deg cm^−2^ dmol^−1^, whereas further decrease to values below −50,000 deg cm^−2^ dmol^−1^ was observed for POC1+POC2+POC3 (Supplementary Fig. 20 and Supplementary Table 1). These observations indicate that the interactions between the ON moieties of POC1+POC2+POC3 induced a singularly higher degree of coiled coil in comparison with the unconjugated peptide. The observed CD signal is noteworthy as negative ellipticity values of such a magnitude to the best of our knowledge have never been reported in a purely aqueous solvent at ambient temperature. To evaluate the contribution of the ON region of POC1+POC2+POC3 to the ellipticity at 222 nm, we measured samples of unconjugated ON1+ON2+ON3 at the same concentrations. In correspondence with the spectra shown in Fig. 3, the signal was in every case ∼10% of that of POC1+POC2+POC3 (Supplementary Table 1). CD temperature denaturation studies provided results in agreement with the ultraviolet-melting studies (Supplementary Discussion and Supplementary Figs 21–24).

### Small-angle X-ray scattering

SAXS analysis was used to provide information on overall structure and oligomeric state of the self-assembled complexes (Table 2a). Performing an indirect Fourier transformation gave the overall physical dimensions of the complexes in solution, while the total scattering provided the oligomeric state of the molecules (see details in Supplementary Methods and Supplementary Figs 25 and 26). Analysis of the data assisted by molecular modelling then provided more detailed solution structures (see below and Supplementary Figs 27–29). SAXS measurements typically require a relatively high concentration; in this study 50 μM of each ON/POC unit was used, while the azidopeptide was measured at 150 μM (3 × 50 μM). POC1+POC2+POC3 was measured at 50 μM, as well as at the lower concentrations of 3.6 and 7.2 μM.

The size of the self-assembled structures and the oligomeric state of ON1+ON2+ON3, azidopeptide and individual POCs corresponded well with the expected values. Both ON1+ON2+ON3 and the azidopeptide formed trimers, the latter with dimensions of the longest axis at ∼7 nm and a cross section at ∼2 nm, almost identical to the CoilV_a_L_d_ and carbohydrate-templated peptide self-assembled structures^26^,^51^. ON1+ON2+ON3 also formed a trimer with a longest axis of ∼7.5 nm and a cross section of ∼2 nm, corresponding with a triple-helical DNA structure.

Studying the POCs individually, it was clear that POC1 formed a homo–dimer, while POC2 and POC3 formed homo-trimers, all with overall lengths of 13 nm, corresponding well with a DNA and peptide moiety. POC1 contains a rather short and rigid triplex-forming pyrimidine oligonucleotide moiety with half of the sequence modified by LNA nucleotides, while POC2 and POC3 contain a substantial proportion of purine nucleotide but no LNA. Some unexpected interactions and secondary structures may be formed for POC1, but not POC2 and POC3, which inhibit trimer formation. Furthermore, a thinner rod was observed for POC1 (short and rigid TFO sequence), which may also contribute to the dimeric appearance of POC1, although the dimensions are shown to be approximately as for POC2/POC3. Furthermore, a small feature was observed for the POCs in the SAXS data around 0.1 Å^−1^ (Supplementary Fig. 25), which arose from the formed two-domain two-contrast structure, again supporting the formation of the expected structure.

For the POC1+POC2+POC3 (1:1:1) combination, SAXS data were acquired at 3.6, 7.2 and 50 μM to study self-assembly at different concentrations (Supplementary Fig. 25). Measuring SAXS at low concentrations can be challenging, but was achieved at the beam line BM-29 at the European Synchrotron Radiation Facility, while POC samples at 50 μM were measured at beamline B21 at the Diamond light Source (see Supplementary Information). Rewardingly, at the low concentrations, scattering corresponding to the POC1+POC2+POC3 trimer was observed. Interestingly, at high concentrations an apparent hexamer, interpreted as a dimer of trimers, assembled as the major component. Despite severe dissociation, both size-exclusion chromatography and gel electrophoresis showed the presence of both trimer and multimers, even at concentrations down to 0.5 μM (Supplementary Figs 17 and 40).

The scattering of the POC1+POC2+POC3 assembly and the pair distance distribution function (Supplementary Figs 25 and 26) showed that at low concentrations (3.6 and 7.2 μM) both the scattering extrapolated to zero angle and the maximum distances (13.0 and 12.7 nm) corresponded with a POC1+POC2+POC3 monomer. In contrast, at high concentration the mass (∼60 kDa) and overall size (∼16.5 nm) corresponded with a dimer of POC1+POC2+POC3. The longest distance was not double the distance found for the individual POCs, which indicated that either the dimer of trimers was not a fully linear structure, or that at least parts of the trimers interact and overlap.

### Molecular modelling

We used molecular modelling to propose detailed POC1+POC2+POC3 models consistent with the SAXS measurements. Our approach (see Supplementary Methods and Supplementary Discussion section) yielded an ensemble of models for which the monomeric POC1+POC2+POC3 structure in Table 2b gave the best fit against the experimental SAXS data at low concentrations (3.6 and 7.2 μM), while the data recorded at 50 μM corresponded very well with the dimer of POC1+POC2+POC3 trimers also shown in Table 2b.

The calculated and experimental SAXS curves showed a very convincing agreement in the experimentally robust 0.01–0.2 Å^−1^ region (see Supplementary Figs 27 and 28). Importantly, the trimer itself can be ruled out as a significant species in solution at the high concentration, due to its characteristic SAXS curve shape deviation in the low *q* range calculated for the MD structures (Supplementary Fig. 28c). Significant amounts of higher-order oligomers, for example, trimers of trimers, can also be excluded since the balancing amount of trimer required to yield the effective molecular weight consistent with SAXS would change the shape of the SAXS curve (Supplementary Figs 28c and 30–31). This point is illustrated with MultiFoXS calculations yielding an inferior curve shape for the composite trimer-of-trimer and trimer curve (Supplementary Fig. 32). A key feature of the POC1+POC2+POC3 dimer of trimers model in Table 2b (Supplementary Fig. 29) is that the two trimers interact hydrophobically via their linkers. This positions Lys residues in one trimer close to the DNA backbone of another, enabling electrostatic interactions between these moieties. The hydrophobic linker interaction was also found for other dimer of trimer models (not shown) with generally low χ (≤20), whereas other types of interaction dominated in dimer of trimers with higher χ, suggesting that the actual solution shape (or ensemble of solution shapes) for the POC1+POC2+POC3 dimer of trimers is well-represented by the model in Table 2b.

### Transmission electron microscopy

**Figure 4:**
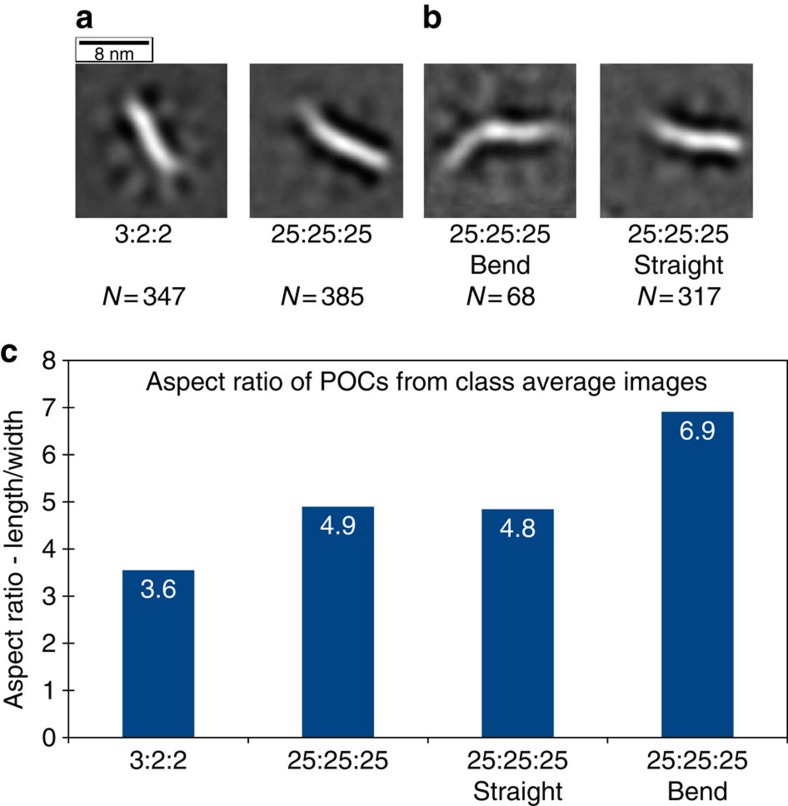
TEM analysis. (**a**) TEM class average images of POC1+POC2+POC3 at low ‘3:2:2' (3 μM in POC1 (TFO) and 2 μM in POC2 and POC3) and high ‘25:25:25' (25 μM in each POC). (**b**) Average images of the two subcategories (straight and bend) at high concentration. (**c**) The length and width was measured of the class average image and the aspect ratio was calculated. TEM class average images are 176 × 176 Å.

The POC1+POC2+POC3 assembly was analysed by TEM (Fig. 4 and Supplementary Fig. 33. See also Supplementary Discussion and Supplementary Methods for details) at two different concentrations, namely ‘3:2:2' (3 μM in POC1 (TFO) and 2 μM in POC2 and POC3) and ‘25:25:25' (25 μM in each POC). The dimensions of the complexes were at the resolution limit of the microscope; hence detailed structural information was not extracted from the data. Class average images of the assemblies show an average higher aspect ratio of the complex at 25 μM than at lower concentration indicative of longer assemblies (Fig. 4). Most of the particles distinguishable at the lower concentration appear to be sized as trimers with a minor proportion appearing as dimerized trimers. In contrast, at 25 μM most of the POCs appear to be dimers of trimers (Fig. 4). It should be noted that drying and staining during the grid preparation procedure could alter the structure, but the increase in aspect ratio in the average images (Fig. 4) and the raw data (Supplementary Figs 34–39) is compatible with a transition from trimers to dimers of trimers at increased concentration. The TEM image analysis also suggests that the POCs have some internal flexibility, especially the 25:25:25, which make the class averaging more difficult. We note that though the TEM data are only indicative, they do give additional support to the observations from gel electrophoretic, CD and SAXS experiments that dimers of trimers dominate at higher concentration and single trimers at lower concentration.

## Discussion

We envisioned that the combination of two fully orthogonal biomolecular self-assembly principles would enable the design of new protein-like structures and higher-order nanoscale-assemblies. Three POCs with molecular weights of 8.5–10.5 kDa were prepared by Cu-free alkyne azide coupling reactions. These POCs formed nano-assemblies which were characterized by ultraviolet thermal denaturation and annealing studies, gel electrophoresis, CD spectroscopy, SAXS and TEM. Ultraviolet and CD melting studies showed that duplexes and triple helices involving POCs are more stable than the non-peptide controls testifying to the importance of interactions between the peptide strands. In contrast, the apparent melting temperatures of the coiled coil peptide remained the same both in triple-helix POCs and as unconjugated peptide. CD spectroscopy further revealed that the triple-helical POC1+POC2+POC3 assembly displayed a high degree of α-helicity, higher than that of the unconjugated peptide. To the best of our knowledge, such α-helicity values have never been reported in a purely aqueous solvent at ambient temperature which points to a helicity-transfer effect from the oligonucleotide triple-helix domain to the coiled coil peptide domain. SAXS performed at high POC concentration revealed the formation of a dimer of heterotrimers of triple-helix POCs, which was corroborated by computational simulation on the ensemble of POCs. At lower concentrations, the obtained SAXS data corresponded to the monomeric POC1+POC2+POC3 assembly. A higher-order assembly was also observed by gel electrophoresis. TEM analysis pointed to two different assembly morphologies, mostly trimers at low concentration and mostly dimers of trimers at high concentration. The structure obtained for the dimer of trimers points towards the possible use of the POC building blocks in creation of larger nanoscale assemblies. The successful construction of a trimeric protein-like structure and its dimer clearly demonstrates the potential of POCs for *de novo* design of proteins, including the potential to control the oligomeric state of peptide self-assemblies.

## Methods

### Synthesis of POC1

ON1-BCN (90 nmol, 0.25 ml, dissolved in Milli-Q water) was added to a solution of azidopeptide (113 nmol) in DMSO (0.8 ml) before H_2_O (0.55 ml) was added. The resulting solution was transferred to a Biotage microwave reaction vial (2 ml) and sealed under an atmosphere of nitrogen. The reaction was carried out on a Biotage Initiator microwave synthesizer at 60 °C for 2 h, whereupon all solvents were removed *in vacuo* and the residue was re-dissolved in Milli-Q water. The synthesis procedure was repeated four times. The four crude solutions (from 360 nmol ON1-BCN) were combined, filtered using a GHP Acrodisc 13 mm syringe filter with 0.45 μm GHP membrane and the solution was heated at 90 °C for 2 min to denature secondary structures before slowly cooling down to room temperature to give a solution of crude product ready for further purification.

### Cyclic preparation protocol for synthesis of POC2 and POC3

ON2-BCN (53 nmol, 0.75 ml) was added into a solution of azidopeptide (66 nmol) in DMSO (0.8 ml) before H_2_O (0.05 ml) was added. The solution was transferred into a Biotage microwave reaction vial (2 ml) and sealed under nitrogen atmosphere. The reaction was carried out on a Biotage Initiator microwave synthesizer at 60 °C for 3 h. After all solvents were removed *in vacuo*, a stepwise wash process was undertaken to separate the desired POC from unreacted ON. The white residue was first rinsed with Milli-Q water (2 × 1 ml) to recover intact ON for next round reaction. The residue was subsequently dissolved in buffer A (2 × 1 ml, 0.025 M Tris-HCl, 0.01 M sodium perchlorate, pH 7.6) to obtain the desired POCs. The synthesis procedure was repeated four times and all crude solutions in buffer A (from 212 nmol ON2-BCN) were combined, filtered using GHP Acrodisc 13 mm syringe filter with 0.45 μm GHP membrane and heated to 90 °C to denature secondary structures before slowly cooling down to room temperature to give a solution of crude product for further purification. The same preparation cycle was applied on synthesis of POC3 (Supplementary Fig. 6): ON3-BCN (48 nmol, 0.735 ml) was added into a solution of azidopeptide (60 nmol) in DMSO (0.735 ml). The synthesis procedure was repeated four times. All crude solutions in buffer A (from 192 nmol ON3-BCN) were combined, filtered using GHP Acrodisc 13 mm syringe filter with 0.45 μm GHP membrane and heated 90 °C to denature secondary structures before slowly cooling down to room temperature to give a solution of crude product for further purification.

### Purification and analysis

The three crude POCs were purified by IE-HPLC using a GE PURIFIER 10 system equipped with a SOURCE 15Q 4.6/100 PE column (15 μm, 100 mm × 4.6 mm). Elution was performed with an isocratic hold of buffer A for 5 min, followed by a linear gradient to 35% buffer B in 45 min at a flow rate of 2.5 ml min^−1^ (buffer A: 0.025 M Tris-HCl, 0.01 M sodium perchlorate, pH 7.6; buffer B: 0.025 M Tris-Cl, 1.0 M sodium perchlorate, pH 7.6). After IE-HPLC purification, the resulting solutions were de-salted by precipitation of the POC products by first adding an aqueous solution of sodium acetate (3 M, 15 μl) followed by addition of cold ethanol (1 ml, 99% w/w; −20 °C). The resulting suspensions were stored at -20 °C for 1 h, and after centrifugation (13,200 r.p.m., 5 min, 4 °C) the supernatants were removed and the pellet further washed with cold ethanol (2 × 1 ml; −20 °C), dried for 30 min under a flow of nitrogen and then dissolved in Milli-Q water (1.0 ml) to give POC1 (108 nmol, 56% in total), POC2 (98 nmol, 46%) and POC3 (76 nmol, 40%), respectively. Mass spectra of POCs were recorded on a Bruker micrOTOF II focus ESI-TOF MS instrument in ES^−^ mode (representative MS in Supplementary Fig. 5). Analytical IE-HPLC traces were recorded on a Merck-Hitachi Lachrom system equipped with a DNAPac PA100 analytical column (13 μm, 250 mm × 4 mm) heated to 60 °C. Elution was performed with an isocratic hold of buffer B (10%), starting from 2 min hold on 2% Buffer A in Milli-Q water, followed by a linear gradient to 30% buffer A in 23 min at a flow rate of 1.1 ml min^−1^ (buffer A: 1.0 M sodium perchlorate; buffer B: 0.25 M Tris-Cl, pH 8.0; representative IE-HPLC traces in Supplementary Fig. 5). Concentrations of purified POCs were determined by ultraviolet absorbance at 260 nm (extinction coefficients in Supplementary Methods).

### Data availability

All relevant data are available from the corresponding authors on request.

## Additional information

**How to cite this article**: Lou, C. *et al*. Peptide–oligonucleotide conjugates as nanoscale building blocks for assembly of an artificial three-helix protein mimic. *Nat. Commun.* 7:12294 doi: 10.1038/ncomms12294 (2016).

## Supplementary Material

Supplementary Information Supplementary Figures 1-40, Supplementary Table 1, Supplementary Discussion, Supplementary Methods and Supplementary References.

## Figures and Tables

**Table 1:**
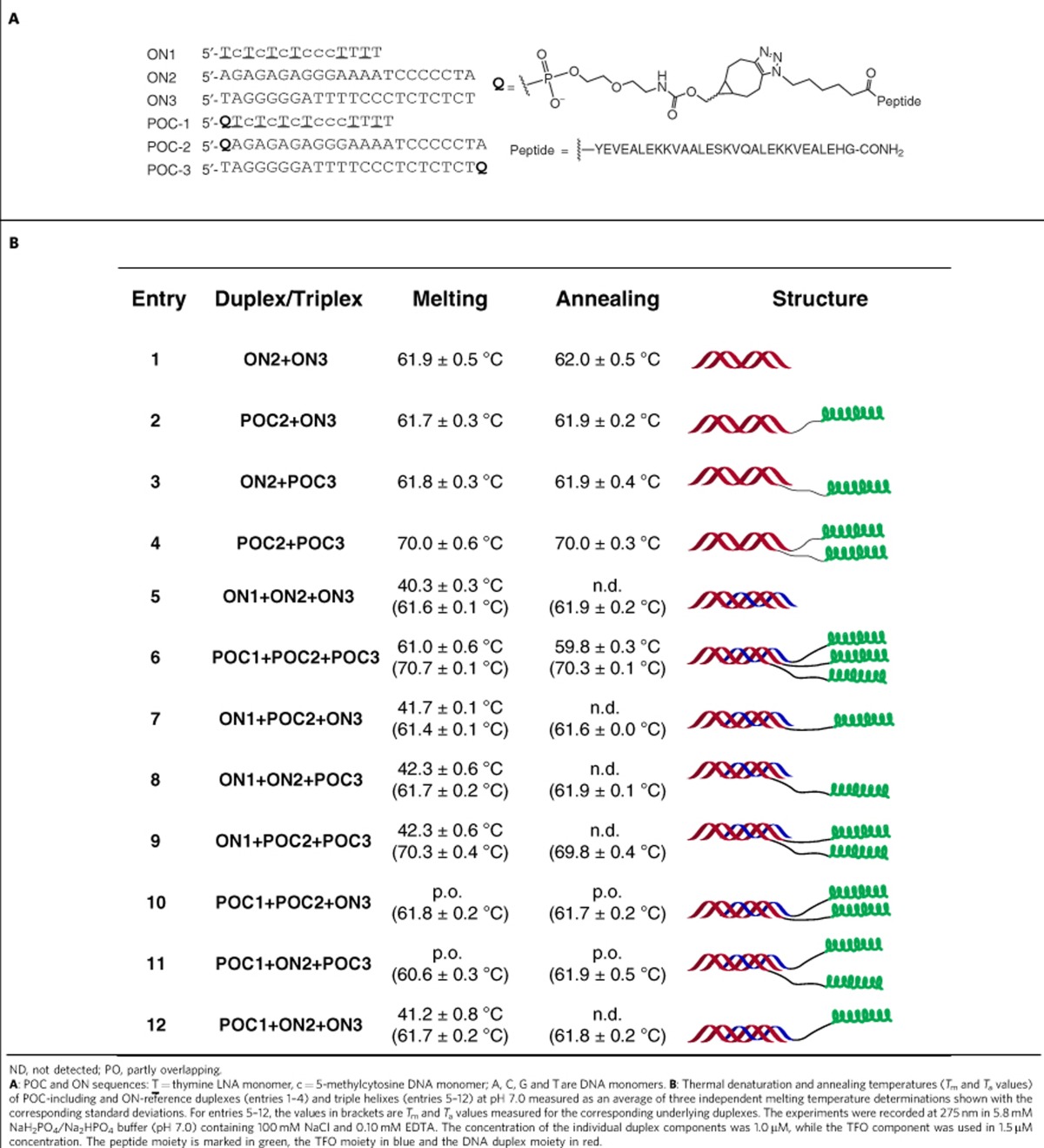
Ultraviolet-melting studies.

**Table 2:**
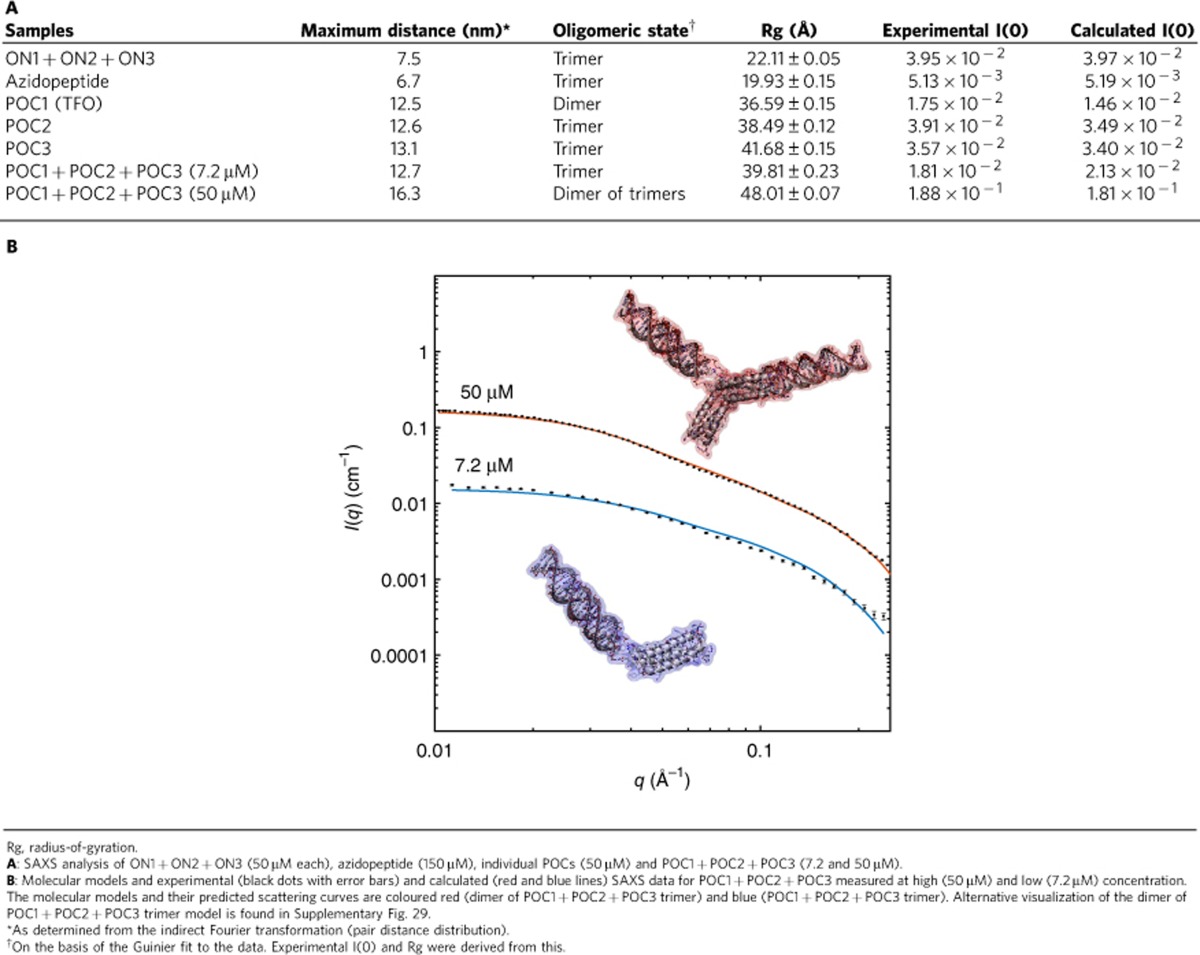
SAXS studies and computational modelling.
