# Supplementary material for: Peptide–oligonucleotide conjugates as nanoscale building blocks for assembly of an artificial three-helix protein mimic
Source: Nat Commun. 2016 Jul 28;7:12294. doi: 10.1038/ncomms12294 (PMC4974474; doi:10.1038/ncomms12294)
Supplement: Supplementary Information — Supplementary Figures 1-40, Supplementary Table 1, Supplementary Discussion, Supplementary Methods and Supplementary References. [file ncomms12294-s1.pdf]

Supplementary Figures

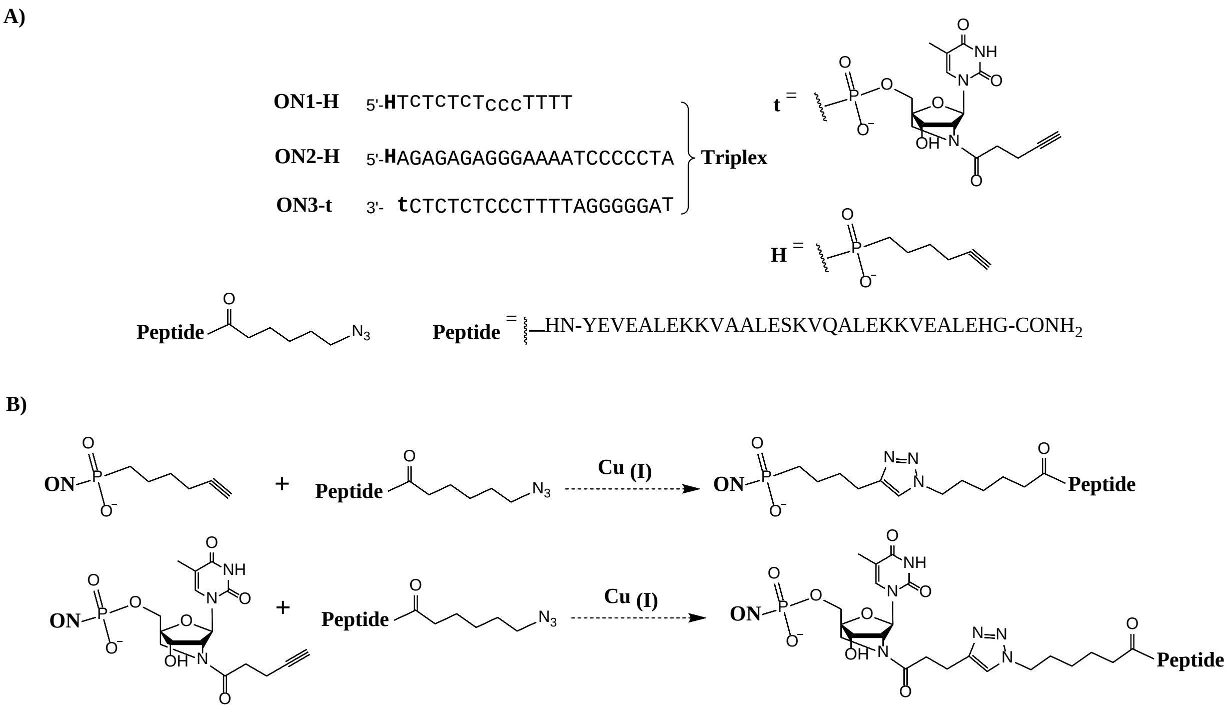

Supplementary Figure 1. Initial design on synthesis of **POC**s from alkyne-labelled oligonucleotides (**ON1-H**, **ON2-H** and **ON3-t**) and **azidopeptide** (30-mer) through Cu(I)-mediated 1,3-dipolar Huisgen cycloaddition. A) Sequence of **Peptide** and triplex formed by **ON1-H**, **ON2-H** and **ON3-t**. B) Proposed Cu(I)-catalyzed azide-alkyne cycloaddition between azido-labelled peptide and three alkyne-containing oligonucleotides.

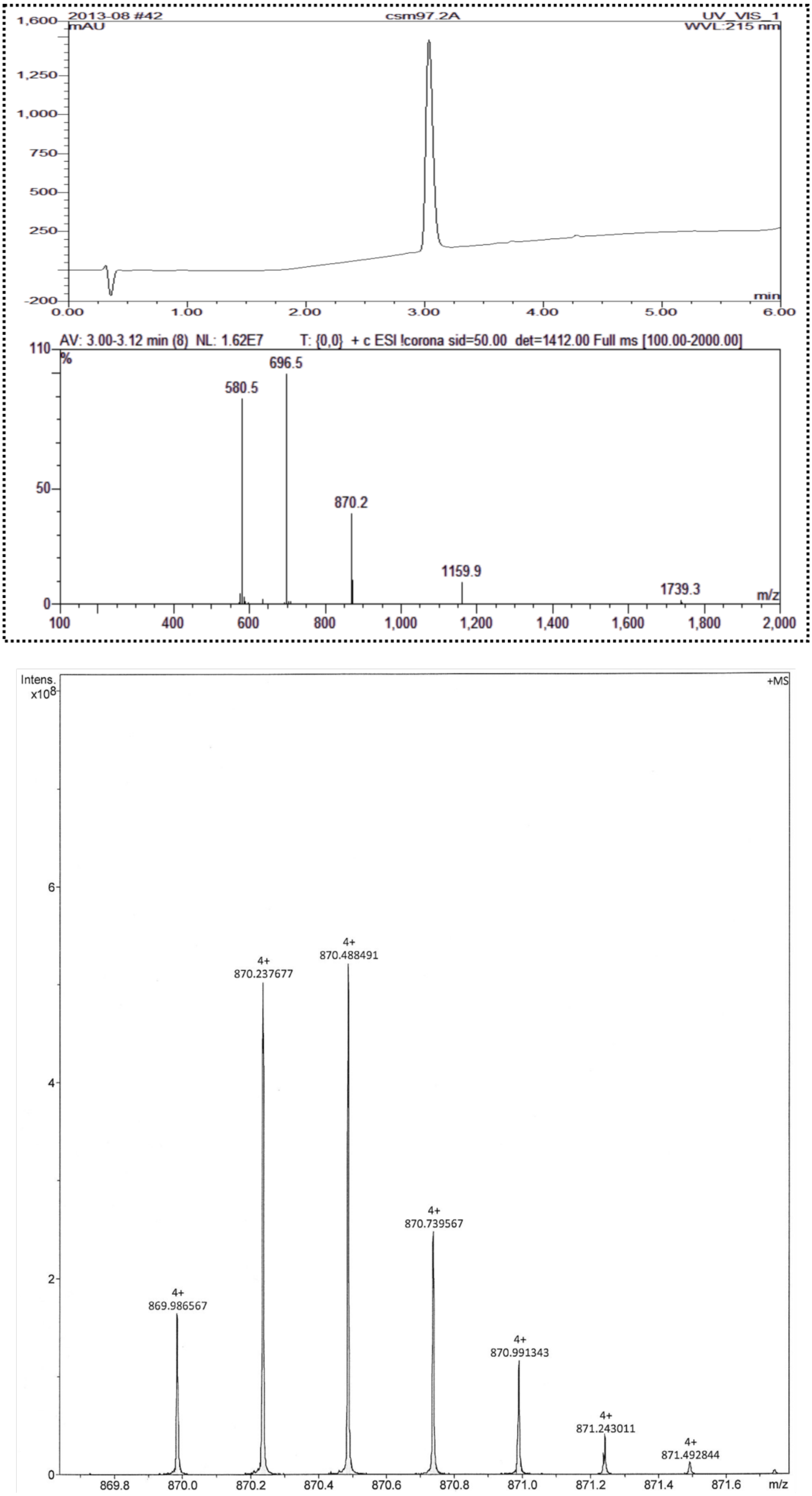

Supplementary Figure 2. LCMS (top) and HRMS (bottom) of **azidopeptide**.

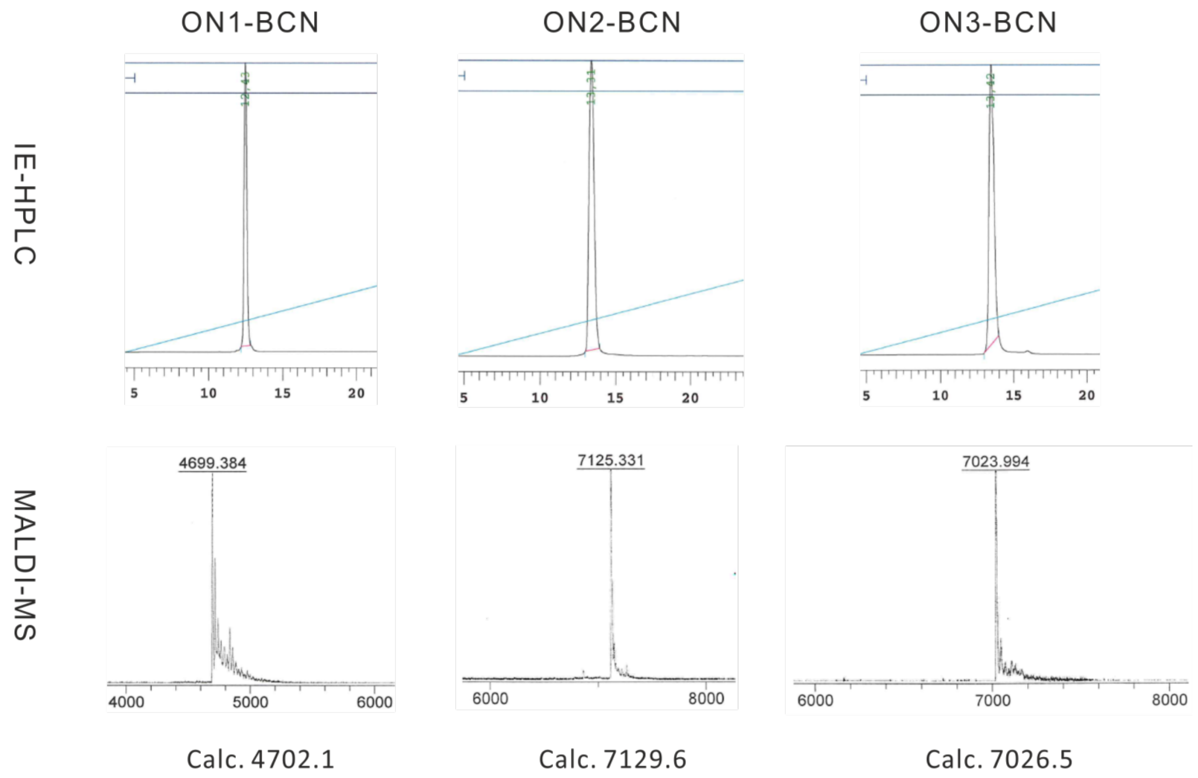

Supplementary Figure 3. Representative analytic IE-HPLC trace and MALDI-MS on **ON1-BCN**, **ON2-BCN** and **ON3-BCN**.

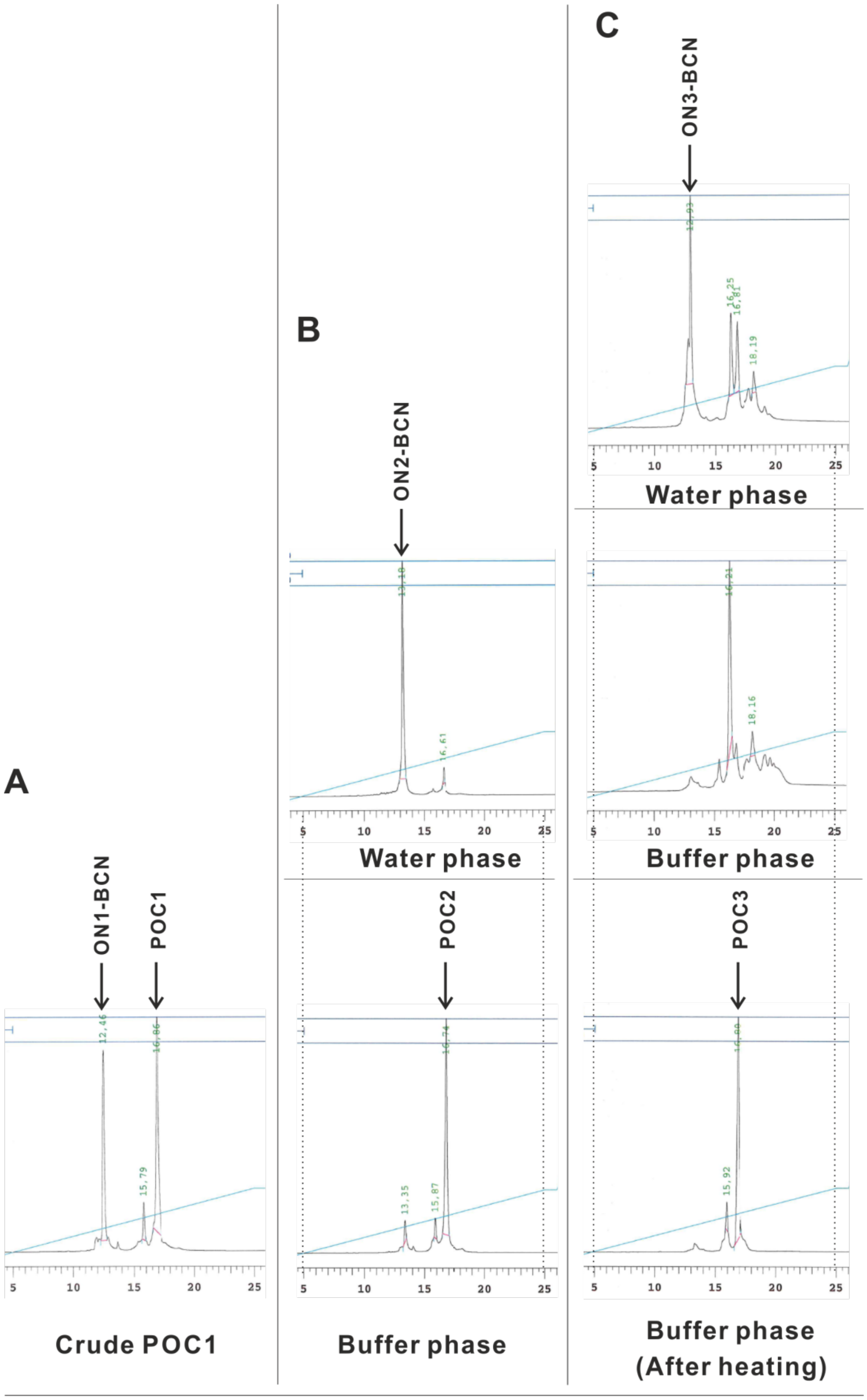

Supplementary Figure 4. Representative IE-HPLC analysis on crude products after microwave reactions: A) Crude **POC1** containing a large amount of unreacted **ON1-BCN**; B) Crude **POC2** (buffer phase) was separated from recycled **ON2-BCN** (water phase); C) crude **POC3** (buffer phase) was separated from recycled **ON3-BCN** (water phase): all possible secondary structures of crude **POC3** were denatured by heating to 90 ˚C and followed by slow cooling down to room temperature.

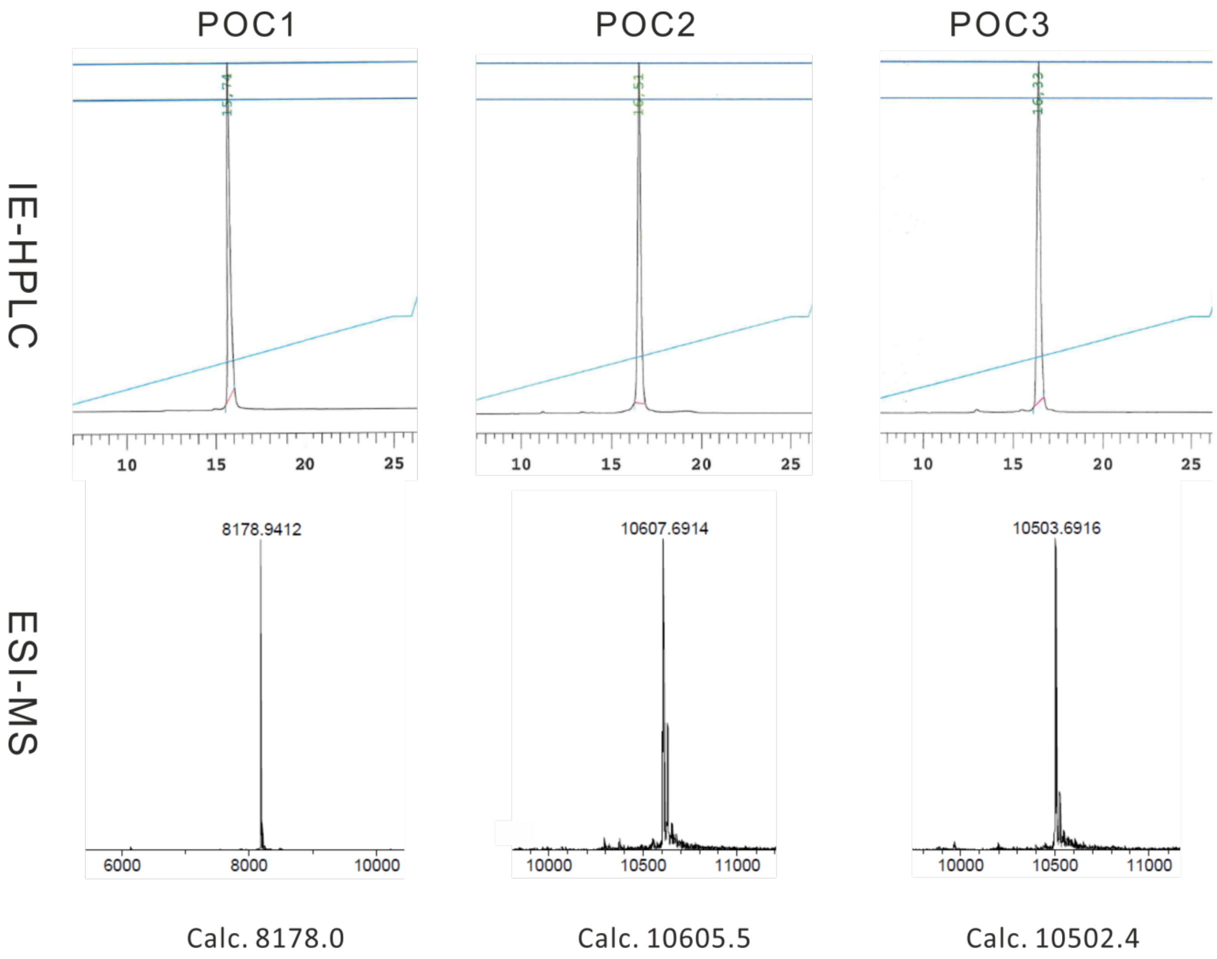

Supplementary Figure 5. Representative analytic IE-HPLC trace and ESI-MS on **POC1**, **POC2** and **POC3**.

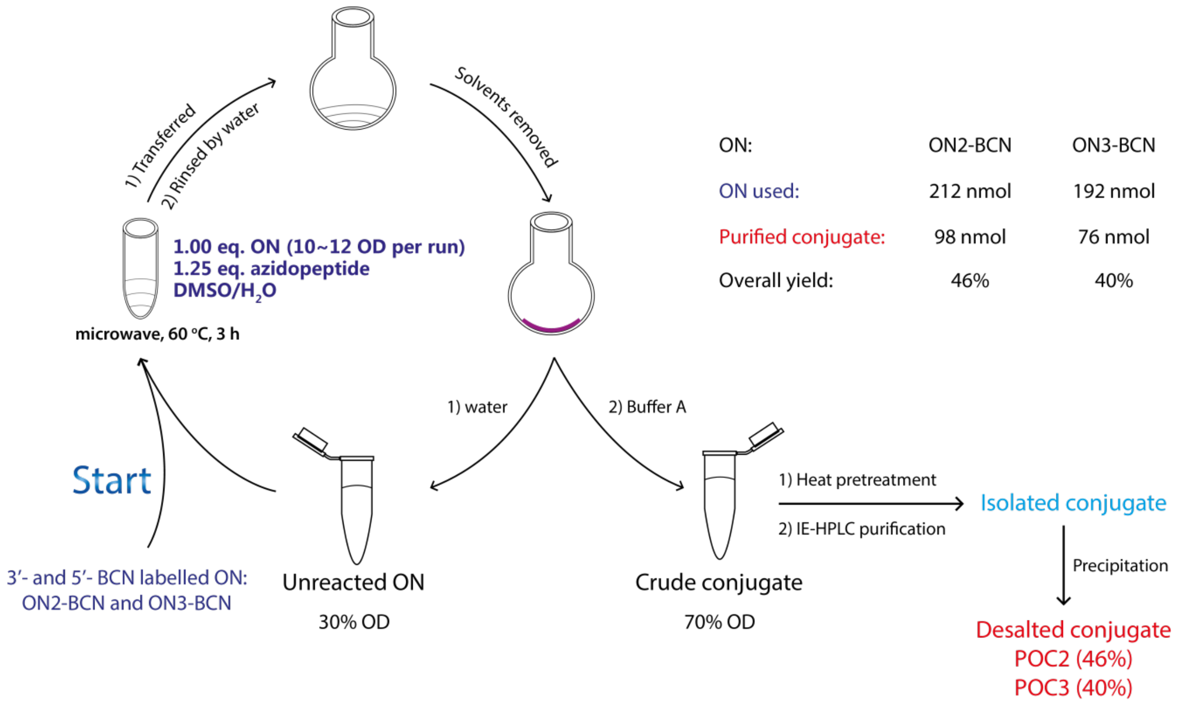

Supplementary Figure 6. Cyclic preparation protocol for synthesis of **POC2** and **POC3**.

5'-TcTcTcTcccTTTT TFO

5'-AGAGAGAGGGAAAATCCCCCTA
3'-TCTCTCTCCCTTTTAGGGGGAT Duplex

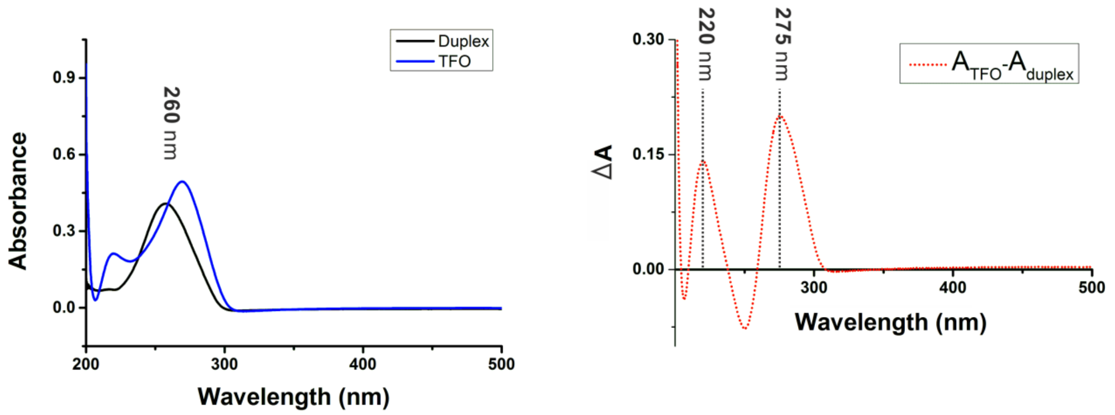

Supplementary Figure 7. UV-scan curves (left) and the absorption difference (right) of TFO (blue, **ON1**) and underlying duplex (black, **ON2**+**ON3**).

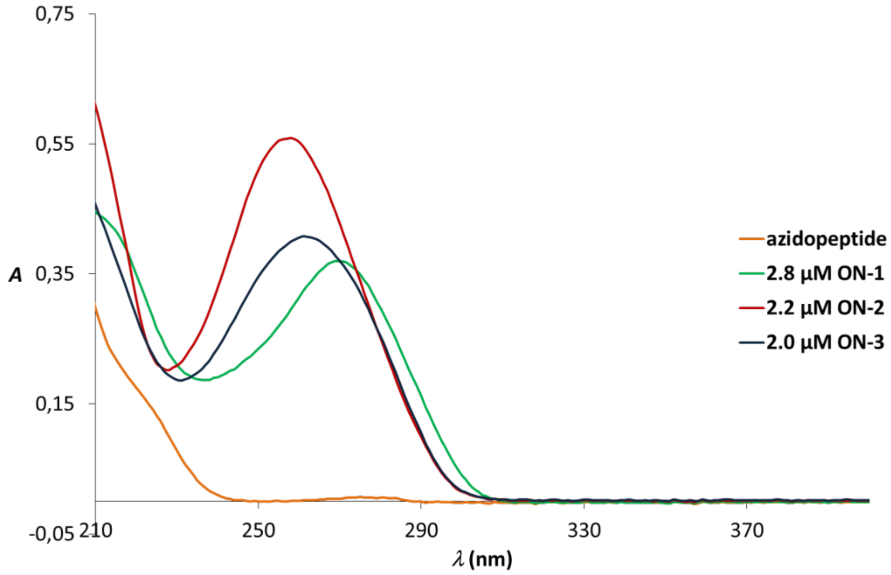

Supplementary Figure 8. UV scan of **azidopeptide** (7 µM), **ON1** (2.8 µM), **ON2** (2.2 µM) and **ON3** (2.0 µM) in 5.83 mM phosphate buffer pH 7.0 with 100 mM NaCl and 0.1 mM EDTA.

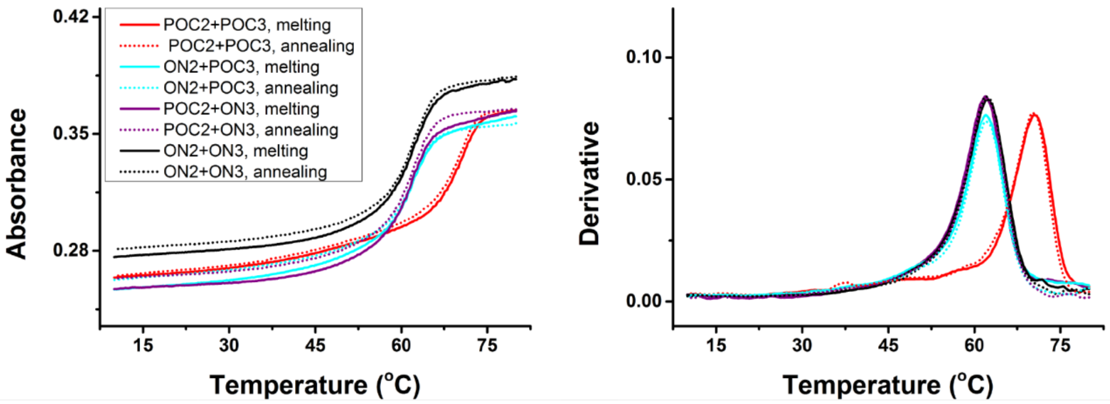

Duplex	Melting	Annealing	Structure
**ON2+ON3**	61.9 ± 0.5 °C	62.0 ± 0.5 °C	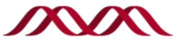
**POC2+ON3**	61.7 ± 0.3 °C	61.9 ± 0.2 °C	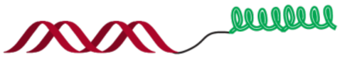
**ON2+POC3**	61.8 ± 0.3 °C	61.9 ± 0.4 °C	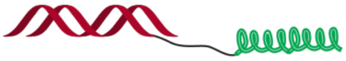
**POC2+POC3**	70.0 ± 0.6 °C	70.0 ± 0.3 °C	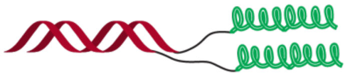

Supplementary Figure 9. UV melting and annealing curves (left) and derivatives (right) of oligo-based, POC-based and hybrid duplexes. T_m and T_a values (°C) are measured as an average of three independent melting and annealing temperature determinations with corresponding standard deviations. The experiments were recorded at 275 nm in 5.8 mM NaH_2PO_4/Na_2HPO_4 buffer (pH 7.0, containing 100 mM NaCl and 0.10 mM EDTA). The concentration of (POC or) oligonucleotide:Watson-Crick complementary strand = 1.0 µM:1.0 µM. The peptide moiety is marked in green and the DNA duplex moiety in red.

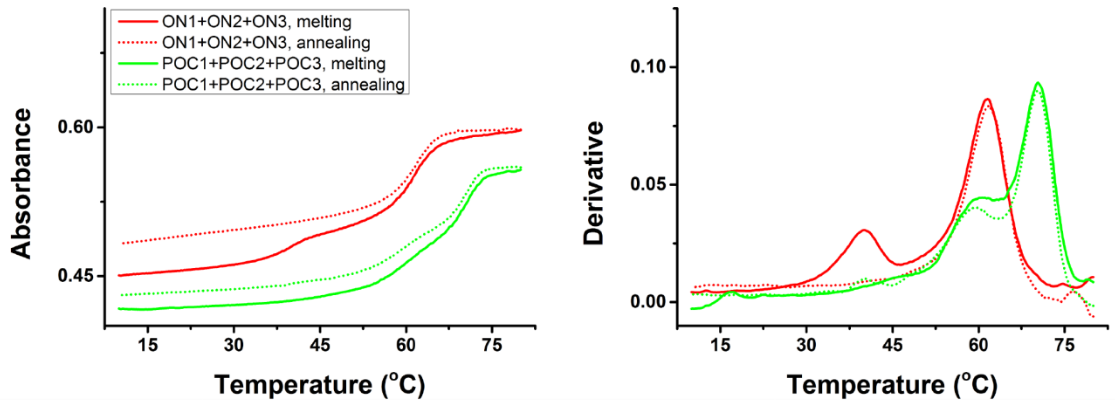

**Triplex**	**ON1+ON2+ON3**	**POC1+POC2+POC3**
**Melting**	40.3 ± 0.3 °C (61.6 ± 0.1 °C)	61.0 ± 0.6 °C (70.7 ± 0.1 °C)
**Annealing**	n.d. (61.9 ± 0.2 °C)	59.8 ± 0.3°C (70.3 ± 0.1 °C)
**Structure**	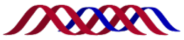	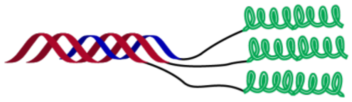

Supplementary Figure 10. UV melting and annealing curves (left) and derivatives (right) of oligonucleotide- and POC-based triplexes. T_m and T_a values (°C) were measured as an average of three independent melting and annealing temperature determinations with corresponding standard deviations. Triplex melting temperatures are given and the values in brackets are T_ms or T_as of corresponding underlying duplexes. The experiments were recorded at 275 nm in 5.8 mM NaH_2PO_4/Na_2HPO_4 buffer (pH 7.0, containing 100 mM NaCl and 0.10 mM EDTA). The concentration of TFO (**POC1** or **ON1**): underlying duplex (**POC2+POC3** or **ON2+ON3**) = 1.5 µM:1.0 µM. The peptide moiety is marked in green. The TFO moiety is in blue and the DNA duplex moiety in red.

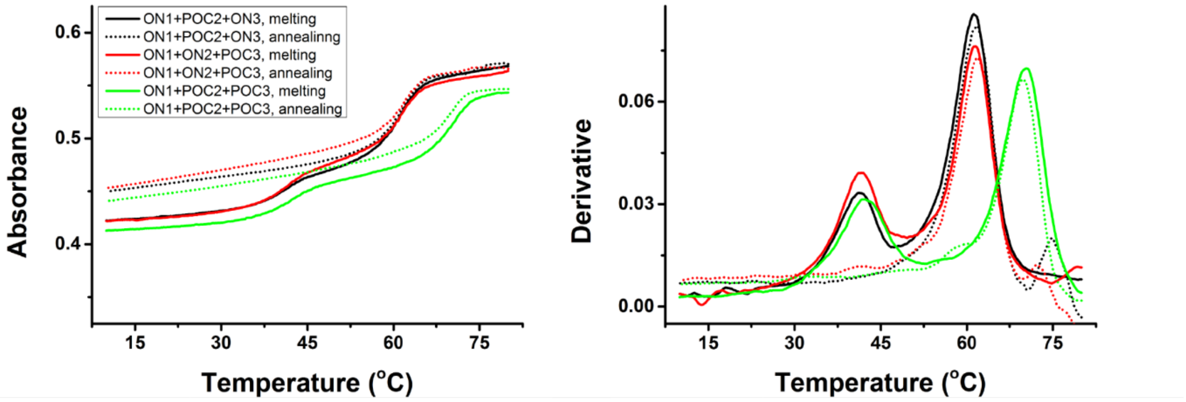

Triplex	Melting	Annealing	Structure
**ON1+POC2+ON3**	41.7 ± 0.1 °C<br>(61.4 ± 0.1 °C)	n.d.<br>(61.6 ± 0.0 °C)	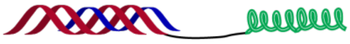
**ON1+ON2+POC3**	42.3 ± 0.6 °C<br>(61.7 ± 0.2 °C)	n.d.<br>(61.9 ± 0.1°C)	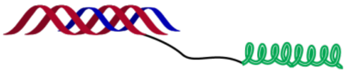
**ON1+POC2+POC3**	42.3 ± 0.6 °C<br>(70.3 ± 0.4 °C)	n.d.<br>(69.8 ± 0.4 °C)	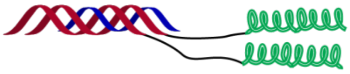

Supplementary Figure 11. UV melting and annealing curves (left) and derivatives (right) of hybrid triplexes (TFO = **ON1**). T_m and T_a values (°C) were measured as an average of three independent melting and annealing temperature determinations with corresponding standard deviations. Triplex melting temperatures are given and the values in brackets are T_ms or T_as of corresponding underlying duplexes. n.d. = not detected. The experiments were recorded at 275 nm in 5.8 mM NaH_2PO_4/Na_2HPO_4 buffer (pH 7.0, containing 100 mM NaCl and 0.10 mM EDTA). The concentration of **ON1**: underlying duplex = 1.5 µM:1.0 µM. The peptide moiety is marked in green. The TFO moiety is in blue and the DNA duplex moiety in red.

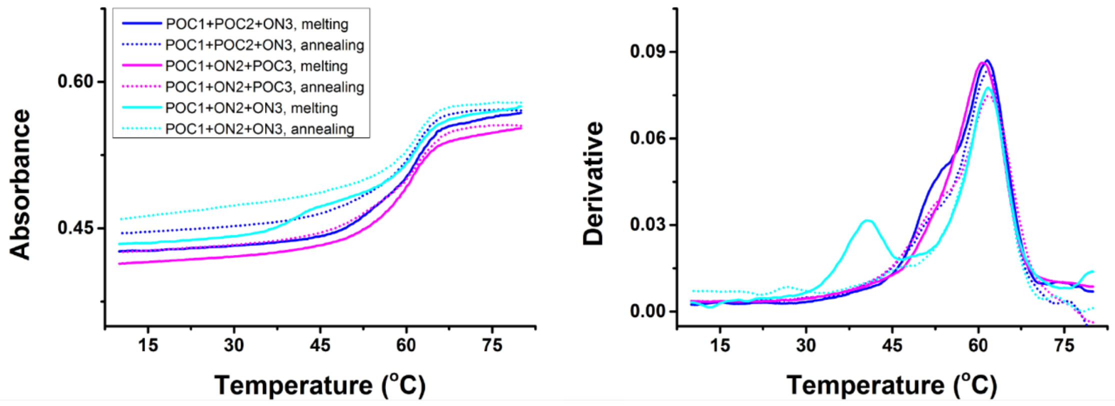

Triplex	Melting	Annealing	Structure
POC1+POC2+ON3	p.o.<br>(61.8 ± 0.2 °C)	p.o.<br>(61.7 ± 0.2 °C)	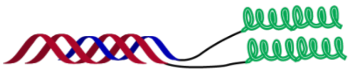
POC1+ON2+POC3	p.o.<br>(60.6 ± 0.3 °C)	p.o.<br>(61.9 ± 0.5°C)	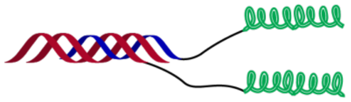
POC1+ON2+ON3	41.2 ± 0.7 °C<br>(61.7 ± 0.2 °C)	n.d.<br>(61.8 ± 0.2 °C)	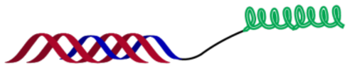

Supplementary Figure 12. UV melting and annealing curves (left) and derivatives (right) of three hybrid triplexes (TFO = **POC1**). T_m and T_a values (°C) were measured as an average of three independent melting and annealing temperature determinations with corresponding standard deviations. Triplex melting temperatures are given and the values in brackets are T_ms or T_as of corresponding underlying duplexes. n.d. = not detected; p.o. = partly overlapped. The experiments were recorded at 275 nm in 5.8 mM NaH_2PO_4/Na_2HPO_4 buffer (pH 7.0, containing 100 mM NaCl and 0.10 mM EDTA). The concentration of **POC1**:underlying duplex = 1.5 µM:1.0 µM. The peptide moiety is marked in green. The TFO moiety is in blue and the DNA duplex moiety in red.

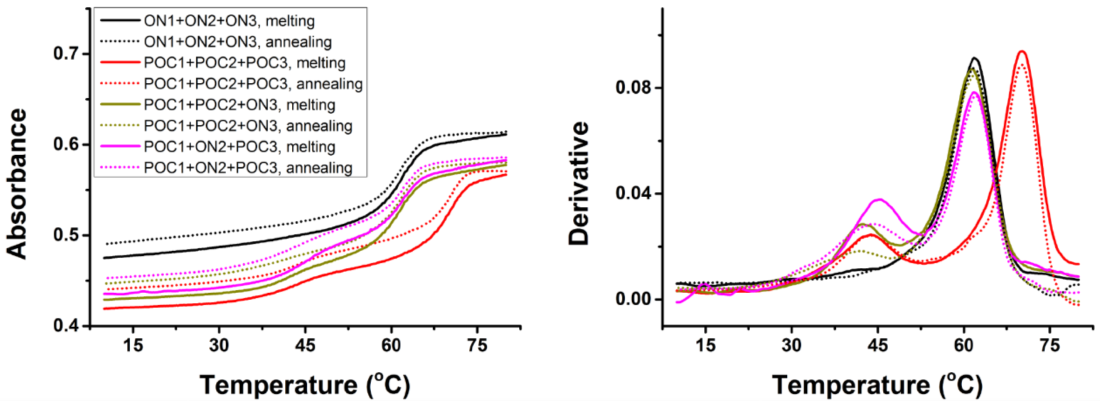

Triplex	Melting	Annealing	Structure
**ON1+ON2+ON3**	n.d. (62.1 ± 0.3 °C)	n.d. (61.7 ± 0.2 °C)	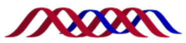
**POC1+POC2+POC3**	43.4 ± 0.6 °C (70.4 ± 0.3 °C)	43.8 ± 0.3 °C (70.3 ± 0.1 °C)	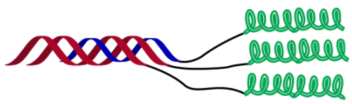
**POC1+POC2+ON3**	42.2 ± 0.4 °C (61.6 ± 0.1°C)	41.7 ± 0.5 °C (61.8 ± 0.2 °C)	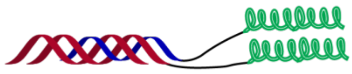
**POC1+ON2+POC3**	46.0 ± 0.6 °C (62.1 ± 0.2 °C)	44.3 ± 0.3 °C (62.2 ± 0.2 °C)	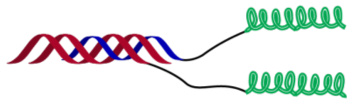

Supplementary Figure 13. UV melting and annealing curves (left) and derivatives (right) of oligonucleotide-based, POC-based and two hybrid triplexes under pH 7.5. T_m and T_a values (°C) were measured as an average of three independent melting and annealing temperature determinations with corresponding standard deviations. Triplex melting temperatures are given and the values in brackets are T_ms or T_as of corresponding underlying duplexes. n.d. = not detected. The experiments were recorded at 275 nm in 5.8 mM NaH_2PO_4/Na_2HPO_4 buffer (pH 7.5, containing 100 mM NaCl and 0.10 mM EDTA). The concentration of **POC1/ON1**:underlying duplex = 1.5 µM:1.0 µM. The peptide moiety is marked in green. The TFO moiety is in blue and the DNA duplex moiety in red.

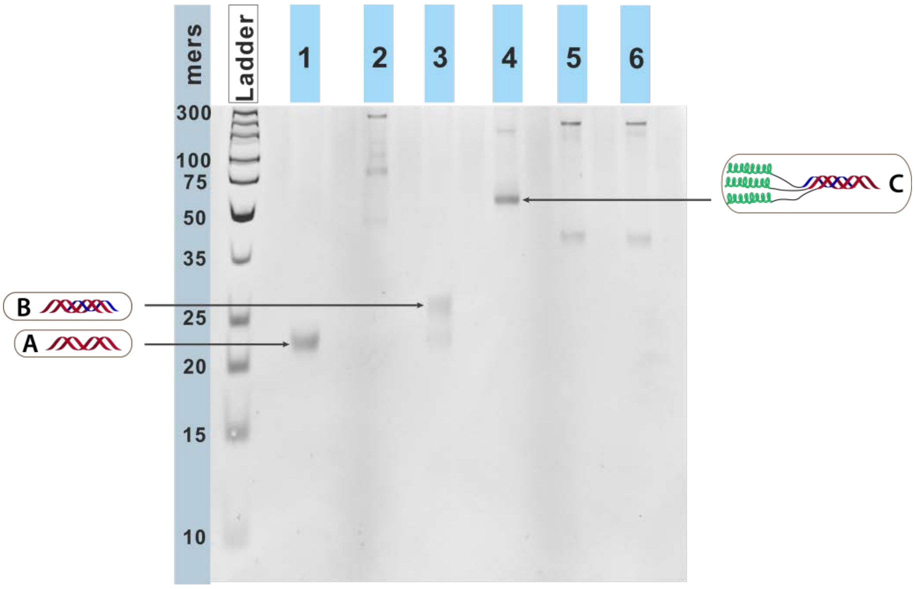

Supplementary Figure 14. 13% PAGE non-denaturing gel at pH 7.0 and room temperature: lane 1, **ON2**+**ON3**; lane 2, **POC2**+**POC3**; lane 3, **ON1**+**ON2**+**ON3**; lane 4, **POC1**+**POC2**+**POC3**; lane 5, **ON2**+**POC3** and lane 6, **POC2**+**ON3**. The gel was visualized by UV excitation at 260 nm after ethidium bromide staining. The O'GeneRuler Ultra Low Range DNA Ladder from bottom to top: 10, 15, 20, 25, 35, 50 75, 100, 150, 200 and 300-mers. The peptide moieties are marked in green, the TFO moiety in blue and the DNA duplex moiety in red.

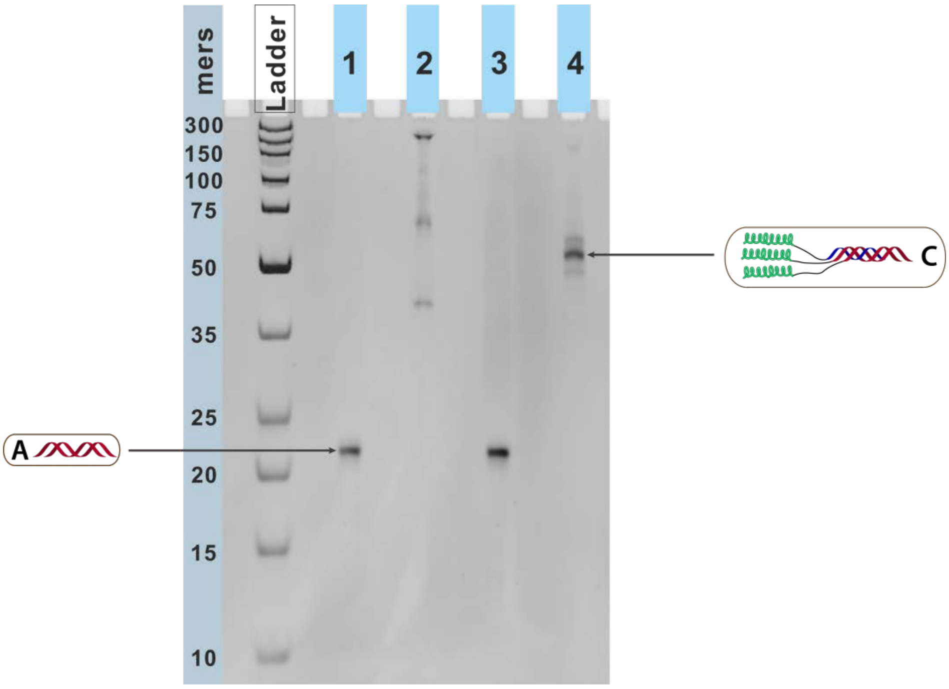

Supplementary Figure 15. 13% PAGE non-denaturing gel at pH 8.5 and 4 °C: lane 1, **ON2**+**ON3**; lane 2, **POC2**+**POC3**; lane 3, **ON1**+**ON2**+**ON3**; lane 4, **POC1**+**POC2**+**POC3**. The gel was visualized by UV excitation at 260 nm after ethidium bromide staining. The O'GeneRuler Ultra Low Range DNA Ladder from bottom to top: 10, 15, 20, 25, 35, 50 75, 100, 150, 200 and 300-mers. The peptide moieties are marked in green, the TFO moiety in blue and the DNA duplex moiety in red.

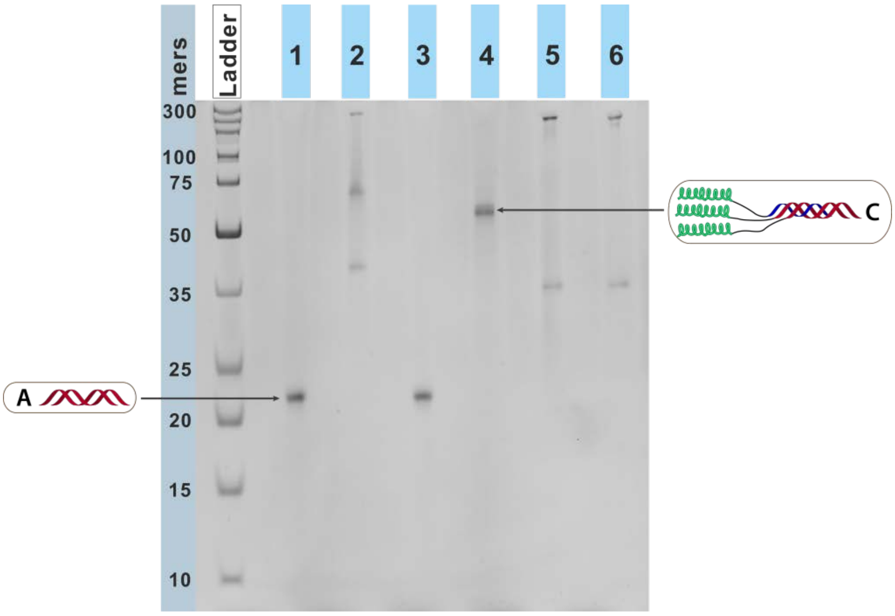

Supplementary Figure 16. 13% PAGE non-denaturing gel at pH 8.5 and room temperature: lane 1, **ON2**+**ON3**; lane 2, **POC2**+**POC3**; lane 3, **ON1**+**ON2**+**ON3**; lane 4, **POC1**+**POC2**+**POC3**; lane 5, **ON2**+**POC3** and lane 6, **POC2**+**ON3**. The gel was visualized by UV excitation at 260 nm after ethidium bromide staining. The O'GeneRuler Ultra Low Range DNA Ladder from bottom to top: 10, 15, 20, 25, 35, 50 75, 100, 150, 200 and 300-mers. The peptide moieties are marked in green, the TFO moiety in blue and the DNA duplex moiety in red.

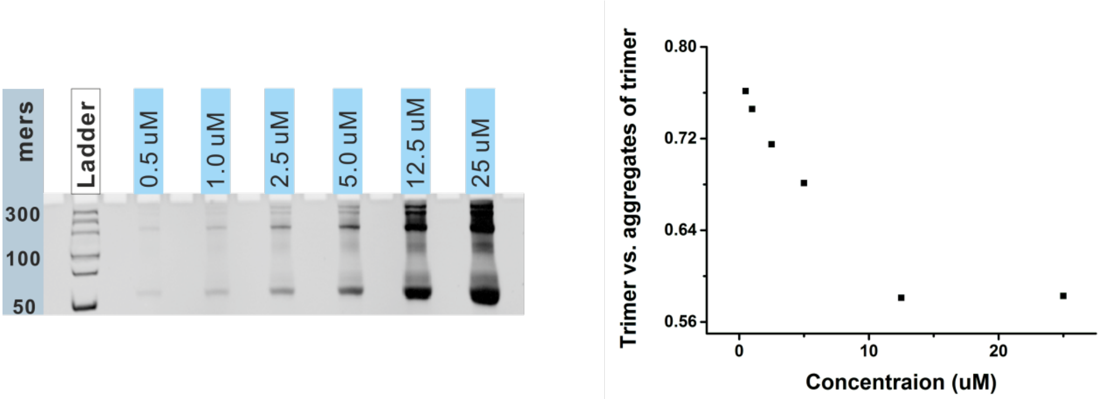

Supplementary Figure 17. Left: 13% PAGE non-denaturing gel of **POC1**+**POC2**+**POC3** (stoichiometry 1:1:1 at different concentrations). The gel was visualized by UV excitation at 260 nm after ethidium bromide staining. The O'GeneRuler Ultra Low Range DNA Ladder from bottom to top: 50 75, 100, 150, 200 and 300-mers. Right: Variation of the ratio of the intensities of the bands corresponding to trimer (lower band) and aggregates of trimer with the concentration from 0.5 uM to 25 uM. Band intensities were integrated and analyzed using imageJ software.

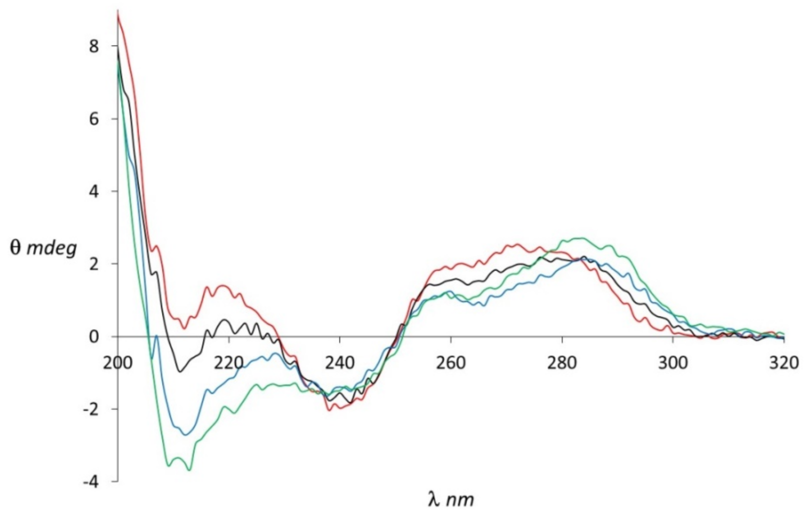

Supplementary Figure 18. Far UV CD spectra of samples containing 2 µM of duplex **ON2**+**ON3** and different concentrations (0 µM = red, 1 µM = black, 2 µM = blue and 3 µM = green) of triplex-forming oligonucleotide strand **ON1**. The spectra were recorded using 0.2 cm path-length cell at 20 ^{o}C in 5.83 mM phosphate buffer pH 7.0 with 100 mM NaCl and 0.1 mM EDTA.

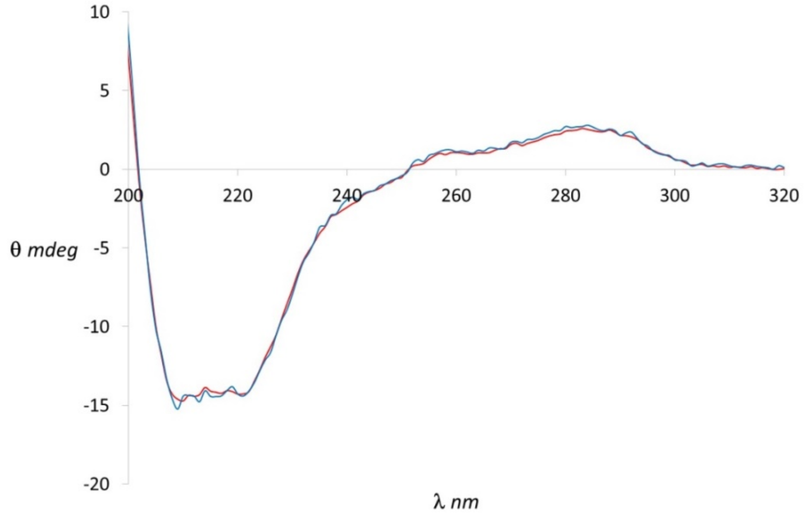

Supplementary Figure 19. Comparison between the far UV CD spectrum of a sample (red) containing **azidopeptide** (7 µM) and triplex ONs (3 µM **ON1 +** 2 µM **ON2** + 2 µM **ON3**) and the mathematical sum (blue) of the individual spectra of **azidopeptide** (7 µM) and **ON1** (3 µM), **ON2** (2 µM) and **ON3** (2 µM) at 20 ^{o}C in 5.83 mM phosphate buffer pH 7.0 with 100 mM NaCl and 0.1 mM EDTA. The spectra were recorded using 0.2 cm path-length cell.

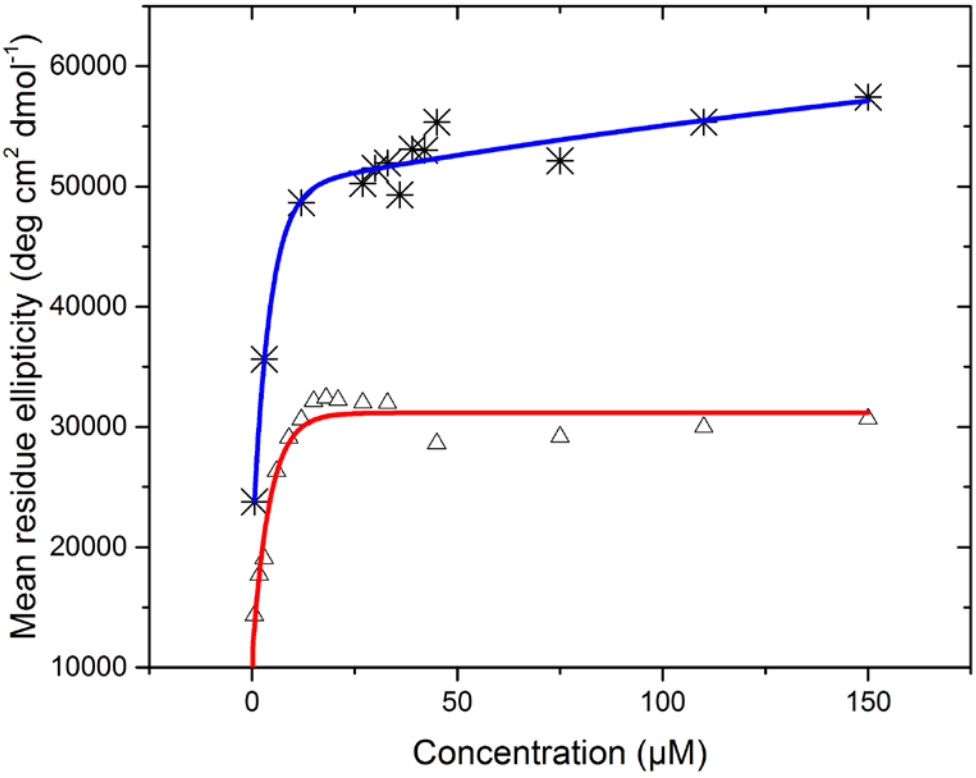

Supplementary Figure 20. Variation of the mean residue ellipticity at 222 nm of **POC1+POC2+POC3** (stoichiometry 1:1:1; blue) and **azidopeptide** (red) with the concentration at 20 ^oC in 5.83 mM phosphate buffer pH 7.0 with 100 mM NaCl and 0.1 mM EDTA.

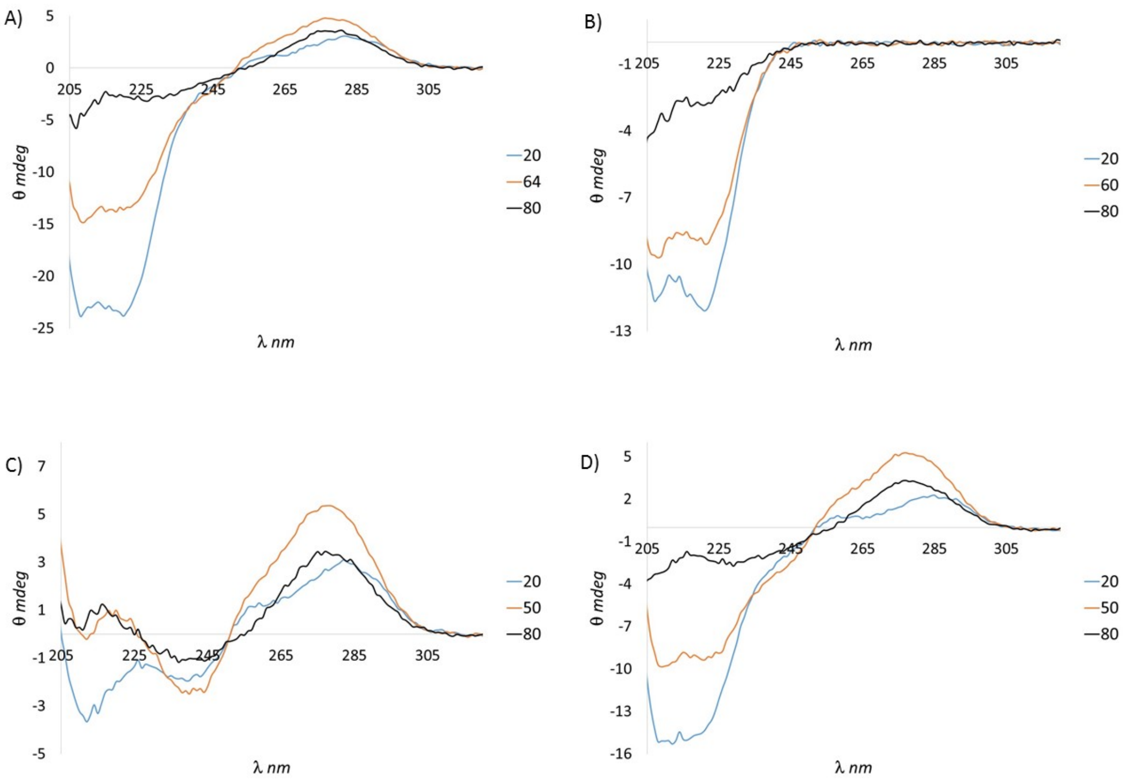

Supplementary Figure 21. Far UV CD spectra recorded at 20 ^oC, 40 ^oC and 80 ^oC. A) 3 µM **POC1** + 2 µM **POC2** + 2 µM **POC3**, B) **azidopeptide** (7 µM), C) triplex oligonucleotides (3 µM **ON1** + 2 µM **ON2** + 2 µM **ON3**) and D) a sample containing **azidopeptide** (7 µM) and triplex oligonucleotides (3 µM **ON1** + 2 µM **ON2** + 2 µM **ON3**) in 5.83 mM phosphate buffer pH 7.0 with 100 mM NaCl and 0.1 mM EDTA.

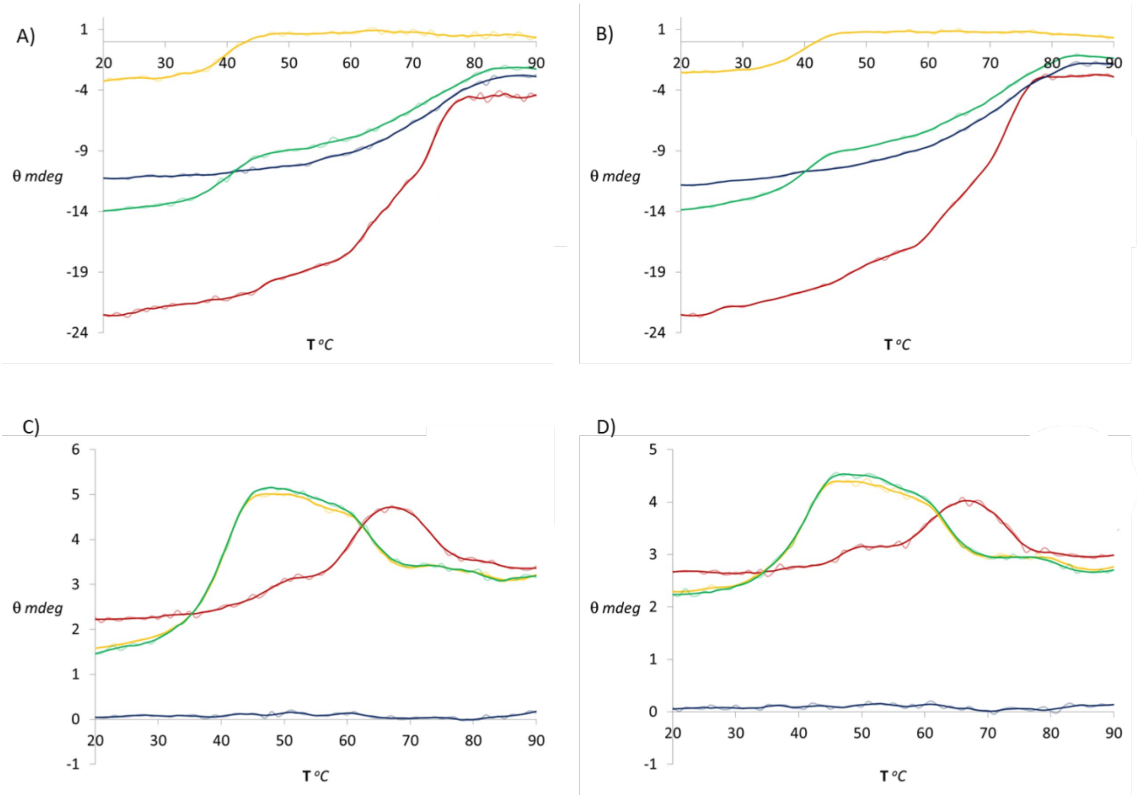

Supplementary Figure 22. CD temperature series. 3 µM **POC1** + 2 µM **POC2 +** 2 µM **POC3** (red), **azidopeptide** (blue; 7 µM), 3 µM **ON1** + 2 µM **ON2** + 2 µM **ON3** (yellow) and a sample containing **azidopeptide** (7 µM) and 3 µM **ON1 +** 2 µM **ON2** + 2 µM **ON3** (green) recorded at 208 nm (A), 220 nm (B), 275 nm (C) and 285 nm (D) in 5.83 mM phosphate buffer pH 7.0 with 100 mM NaCl and 0.1 mM EDTA.

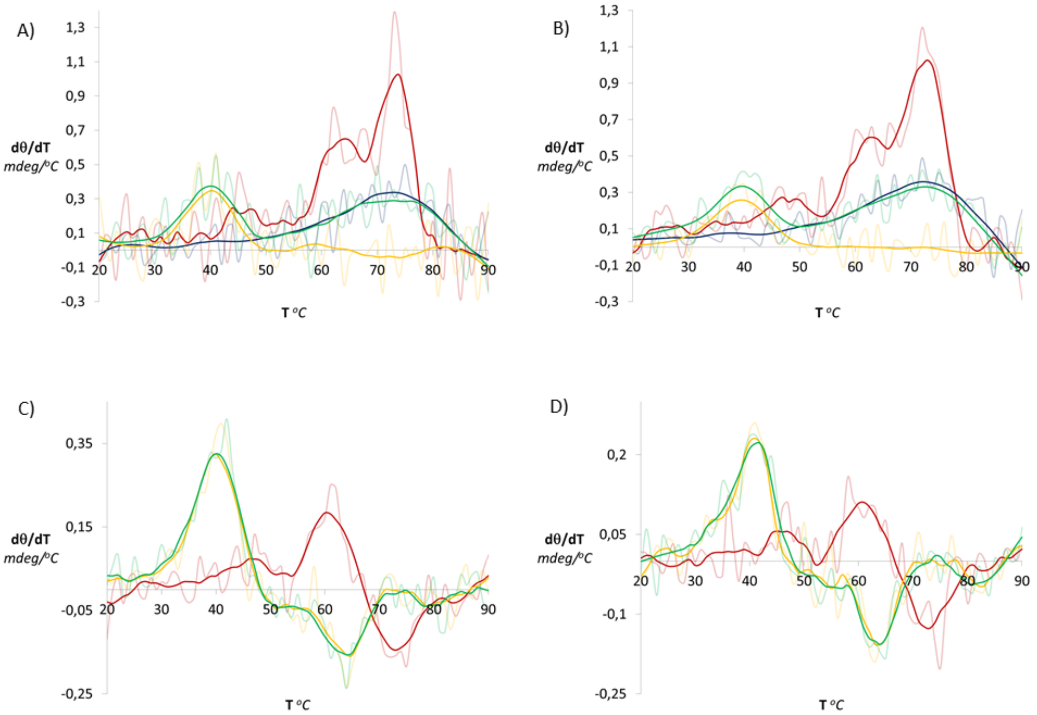

Supplementary Figure 23. First derivatives of the CD temperature series. 3 µM **POC1** + 2 µM **POC2 +** 2 µM **POC3** (red), **azidopeptide** (blue; 7 µM), 3 µM **ON1** + 2 µM **ON2** + 2 µM **ON3** (yellow) and a sample containing **azidopeptide** (7 µM) and 3 µM **ON1 +** 2 µM **ON2** + 2 µM **ON3** (green) recorded at 208 nm (A), 220 nm (B), 275 nm (C) and 285 nm (D) in 5.83 mM phosphate buffer pH 7.0 with 100 mM NaCl and 0.1 mM EDTA.

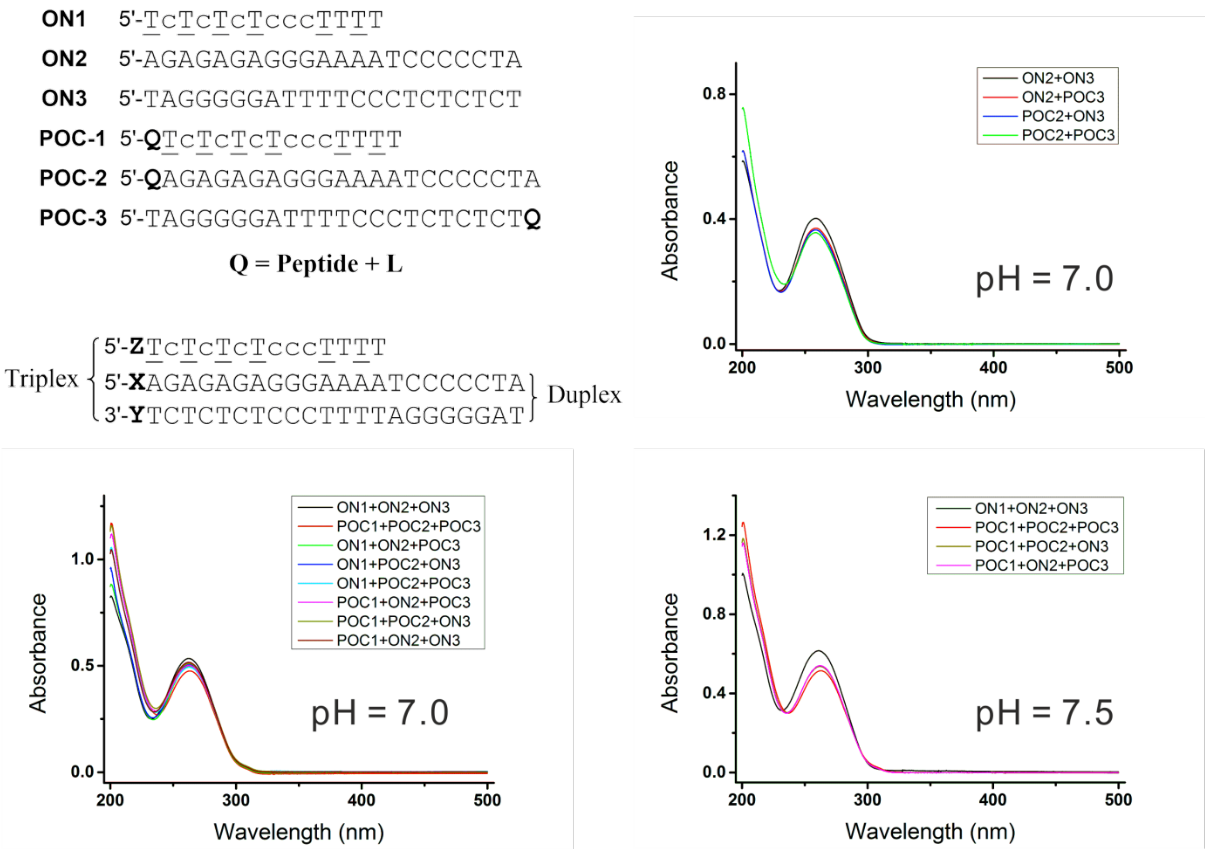

Supplementary Figure 24. UV-scan curves of samples of twelve sequence contexts including four duplexes (upper right) and eight triplexes (down left and right) in 5.8 mM NaH_2PO_4/Na_2HPO_4 buffer (pH 7.0 or pH 7.5, containing 100 mM NaCl and 0.10 mM EDTA). All samples were prepared as described in Supplementary Methods, and used directly for the melting studies.

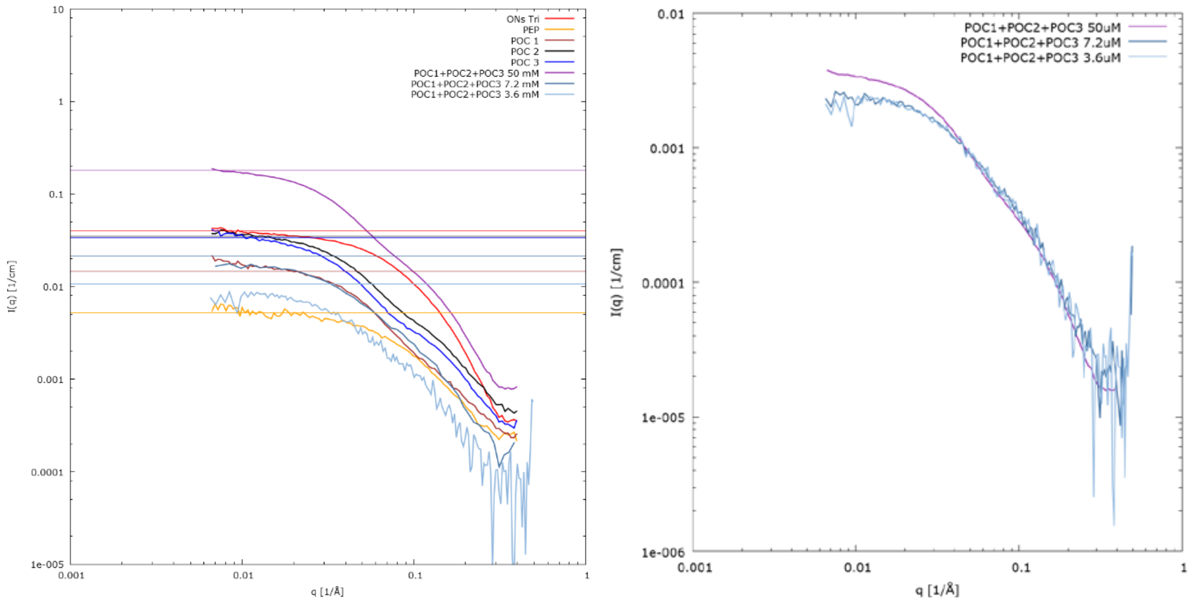

Supplementary Figure 25. Raw SAXS data. Left, all data on absolute scale with the corresponding calculated I(0) values. Right, the three **POC1+POC2+POC3** datasets scaled by concentration.

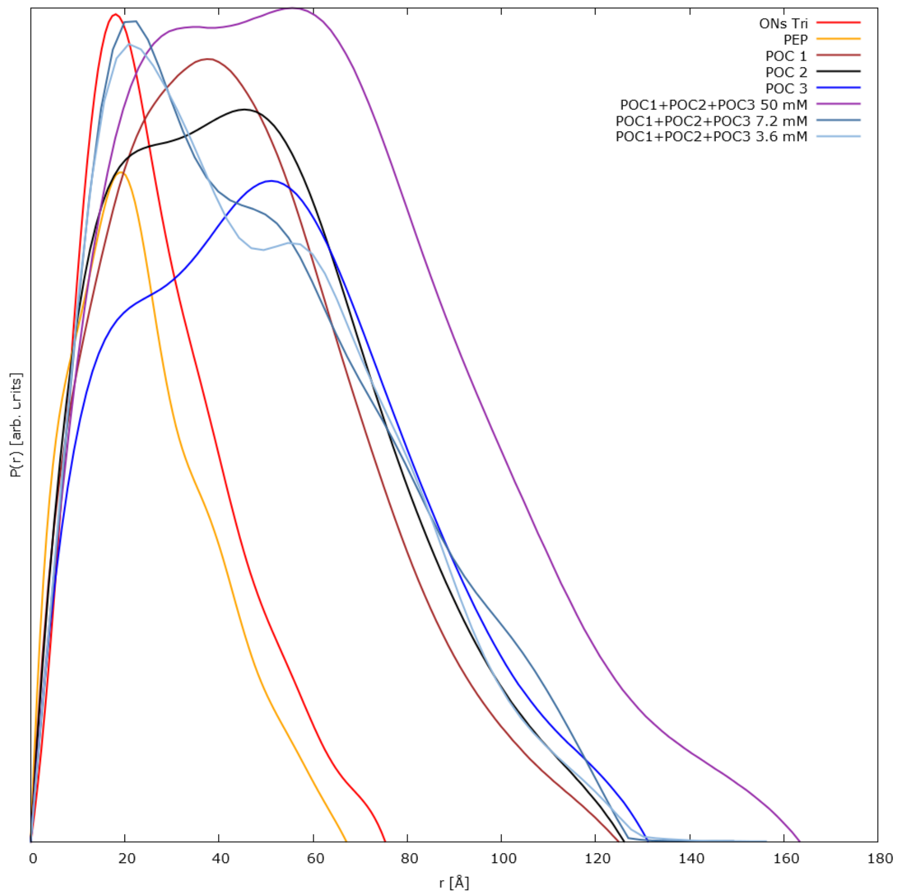

Supplementary Figure 26. Fourier transformed SAXS data, showing the pair-distance distribution contained in the sample.

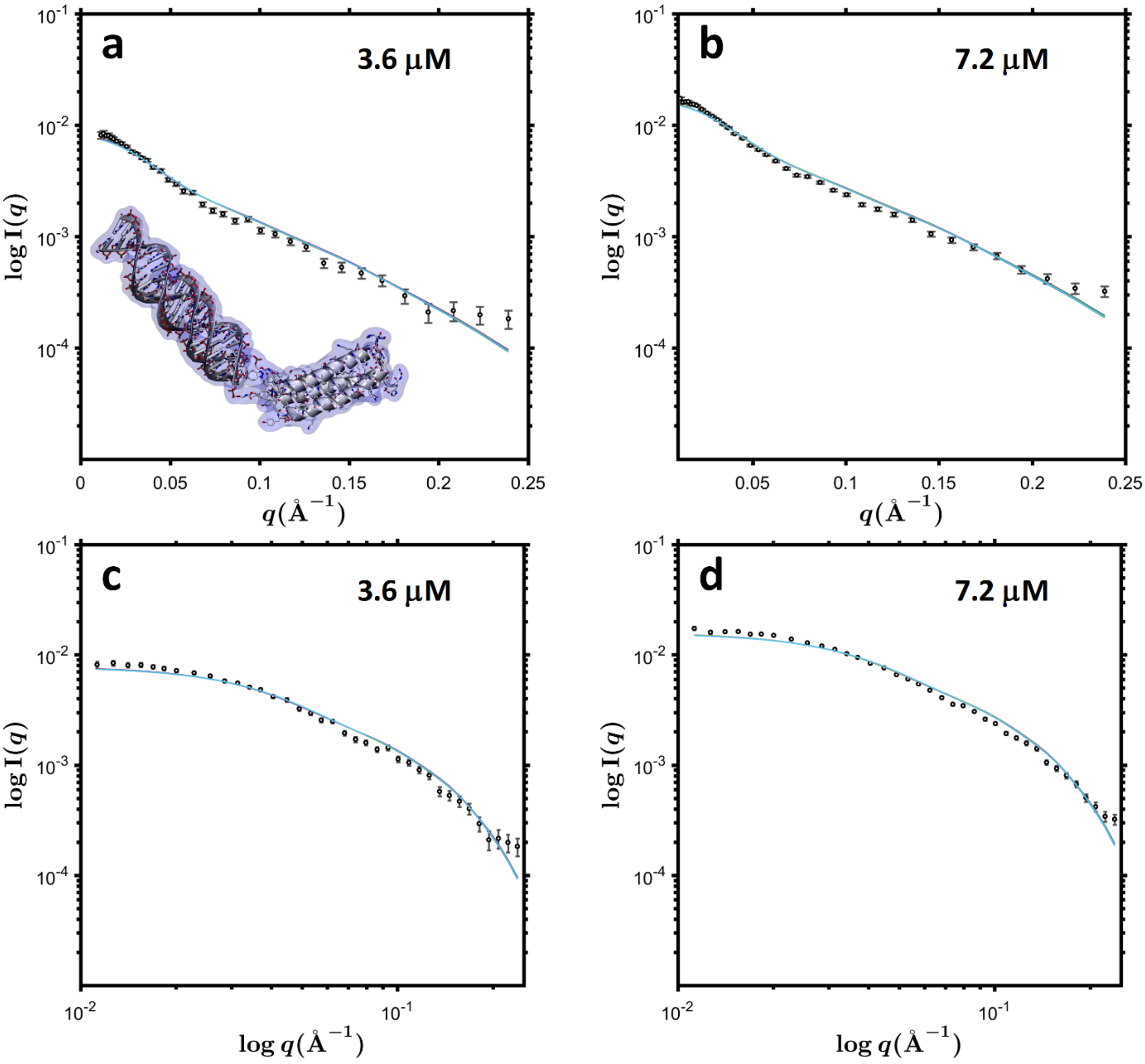

Supplementary Figure 27. SAXS scattering curves from experiment (black dots with error bars) and molecular modelling (colored) for **POC1**+**POC2**+**POC3** measured at the low concentrations 3.6 µM (A, C) and 7.2 µM (B, D). (A) and (B) are semilog plots while (C) and (D) are log-log plots of the same data. The molecular model of the **POC1**+**POC2**+**POC3** trimer giving the best fit to experiment at both 3.6 µM and 7.2 µM is shown inset in (A). Calculated scattering curves for the best fitting 20 conformations of this model are shown in all subfigures and are highly similar, giving the impression of a single curve.

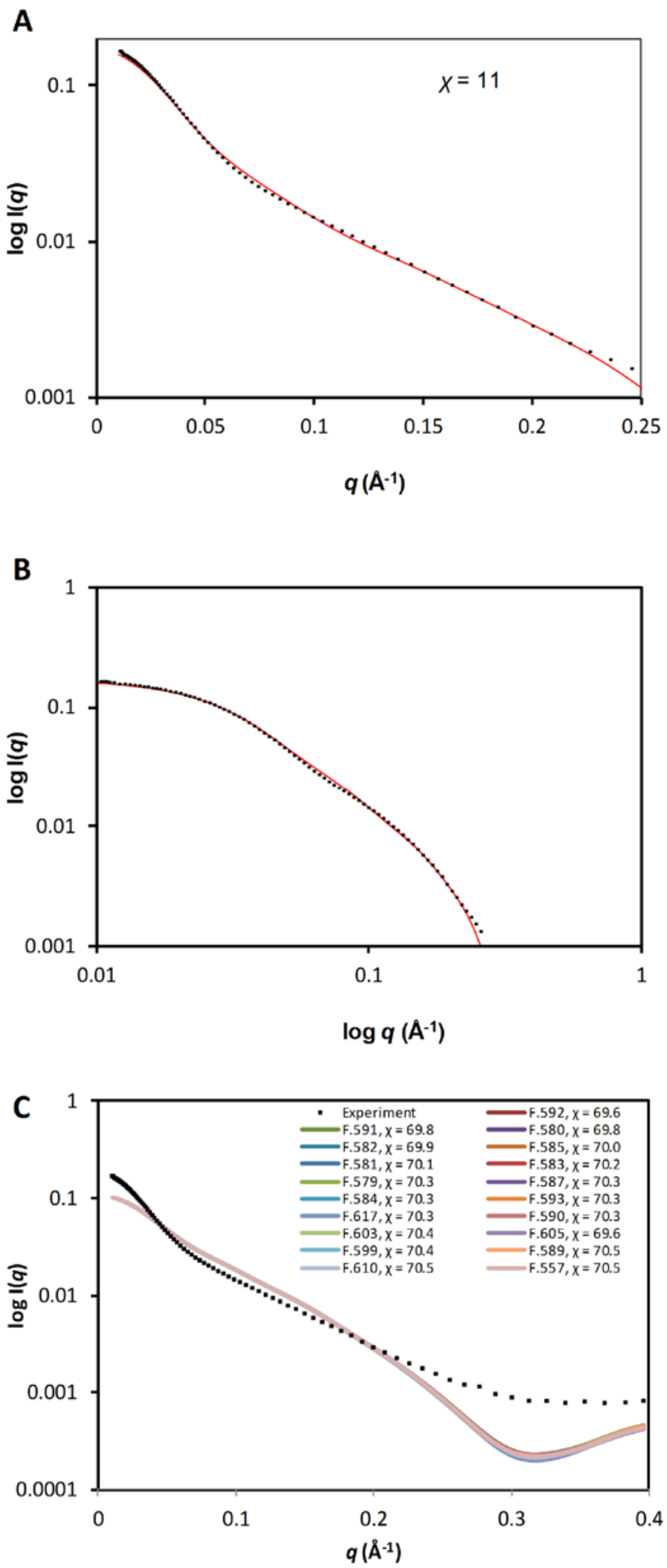

Supplementary Figure 28. SAXS scattering curves from experiment (black dots) and molecular modelling (colored) for **POC1+POC2+POC3** measured at high concentration (50 µM). (A) calculated scattering curve for the best **POC1+POC2+POC3** model (Table 2b, and Supplementary Figure 29). (B) log $I(q)$ vs. log (q) corresponding to (A). (C) sanity check: SAXS curves calculated for 20 MD snapshots of the **POC1+POC2+POC3** trimer model giving the lowest χ upon fitting to the experimental SAXS curve for the **POC1+POC2+POC3** dimer of trimers.

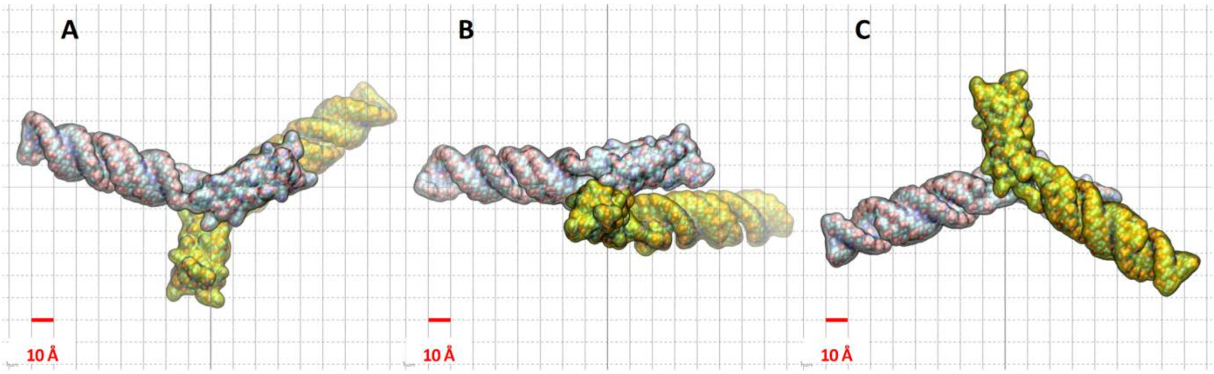

Supplementary Figure 29. Molecular model of the **POC1**+**POC2**+**POC3** dimer of trimers giving the best agreement with SAXS data at high concentration (50 µM) shown from different angles. (**A**) viewed as in the article (Table 2b). (B) rotated 90° around the x axis. (C) rotated 180° around the x axis. One trimer is shown in lime-green color and the other trimer in blue-purple color.

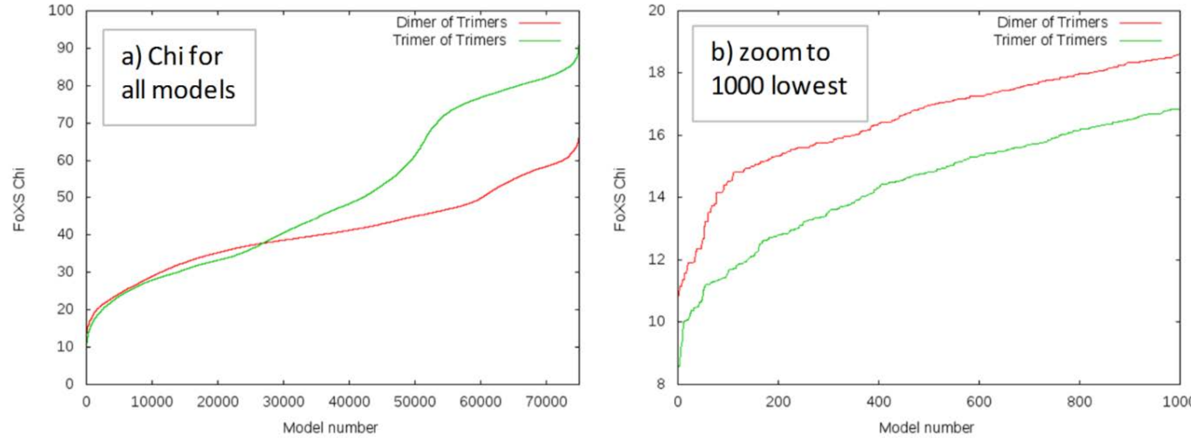

Supplementary Figure 30. FoXS chi (χ) values for 75000 symmetric models of dimers of trimers and trimers of trimers produced by SymmDock. χ-values were calculated for a fit against the experimental SAXS data in the range of 0.010 to 0.255 Å^{-1}.

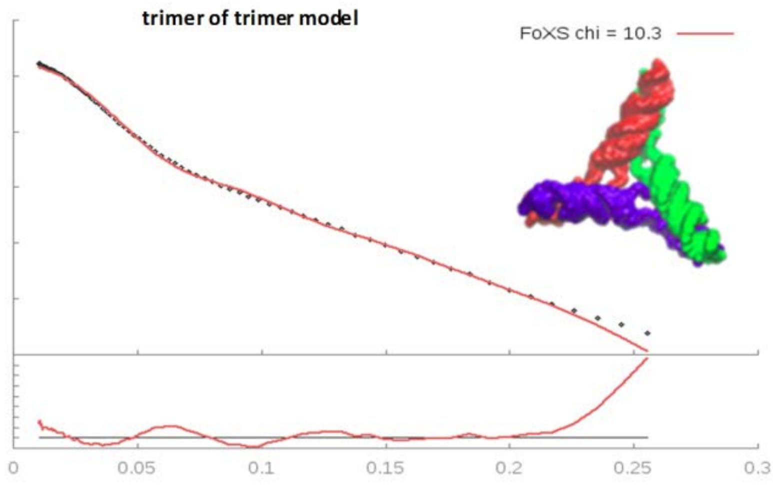

Supplementary Figure 31. Example symmetric trimer **POC1+POC2+POC3** model approximating the shape of the experimental SAXS data in the range 0.010 to 0.255 Å^{-1}.

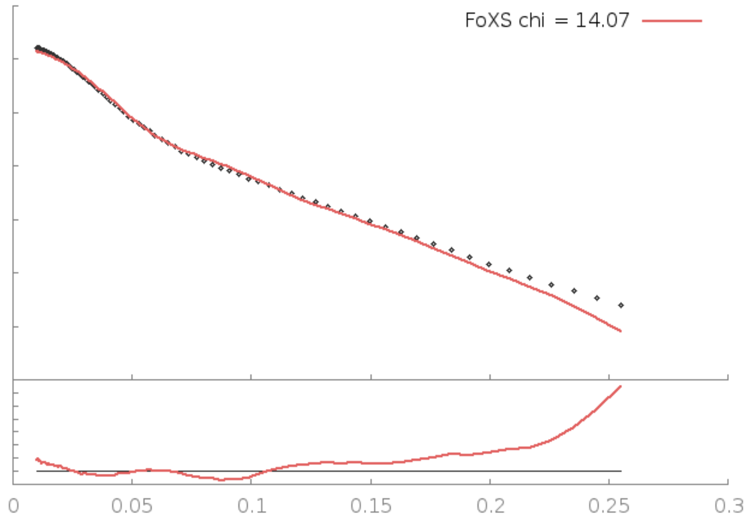

Supplementary Figure 32. Best SAXS curve calculated by MultiFoXS combining a monomeric **POC1+POC2+POC3** model and a trimeric **POC1+POC2+POC3** model.

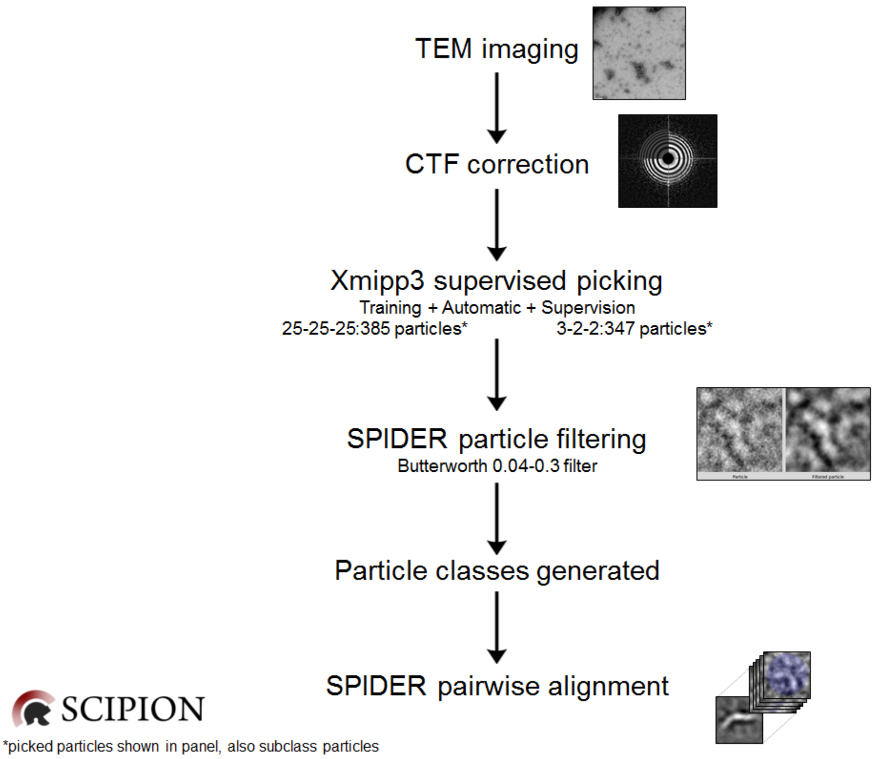

Supplementary Figure 33. Scipion image processing work flow. The TEM images was CTF corrected followed by supervised picking at a high threshold followed by particle filtering, generation of classes and lastly a pairwise alignment.

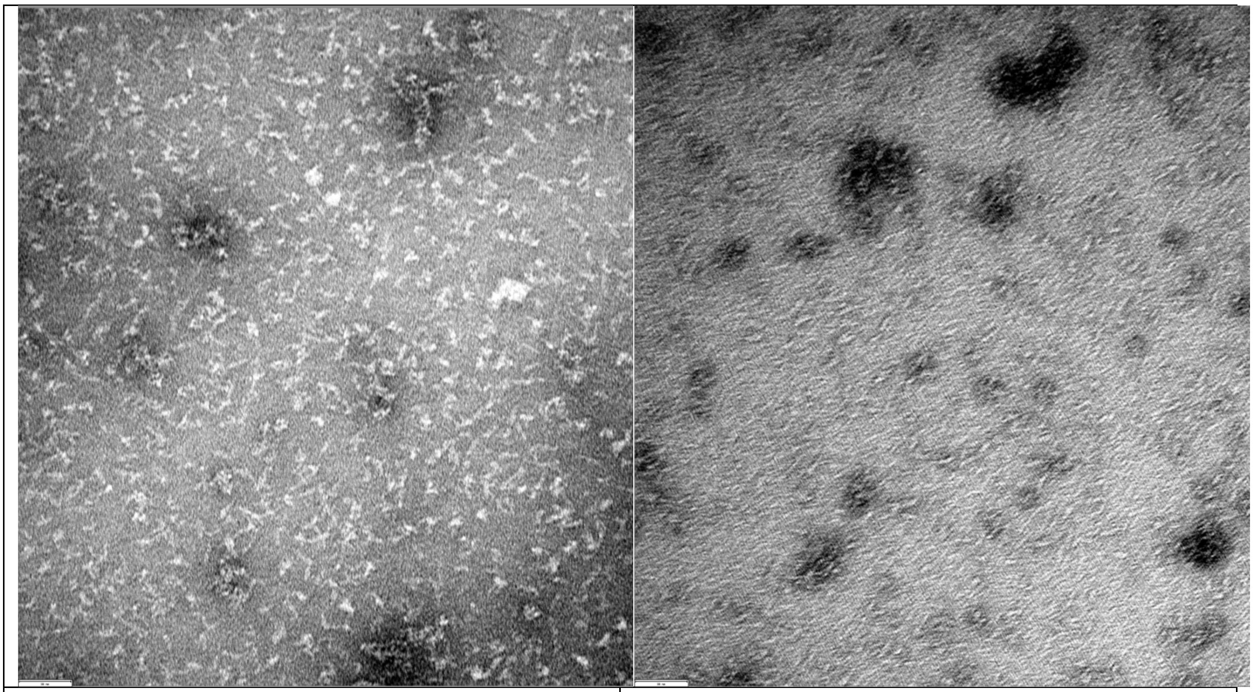

a) 3:2:2 raw image
b) 25:25:25 raw image

Supplementary Figure 34. Raw negative stain TEM images of the TEM grid with the POC triplex assembly (**POC1**+**POC2**+**POC3)** in concentrations of **a**) 3:2:2 µM and **b**) 25:25:25 µM. The scale bar on both images is 30 nm and the images acquired at x110k.

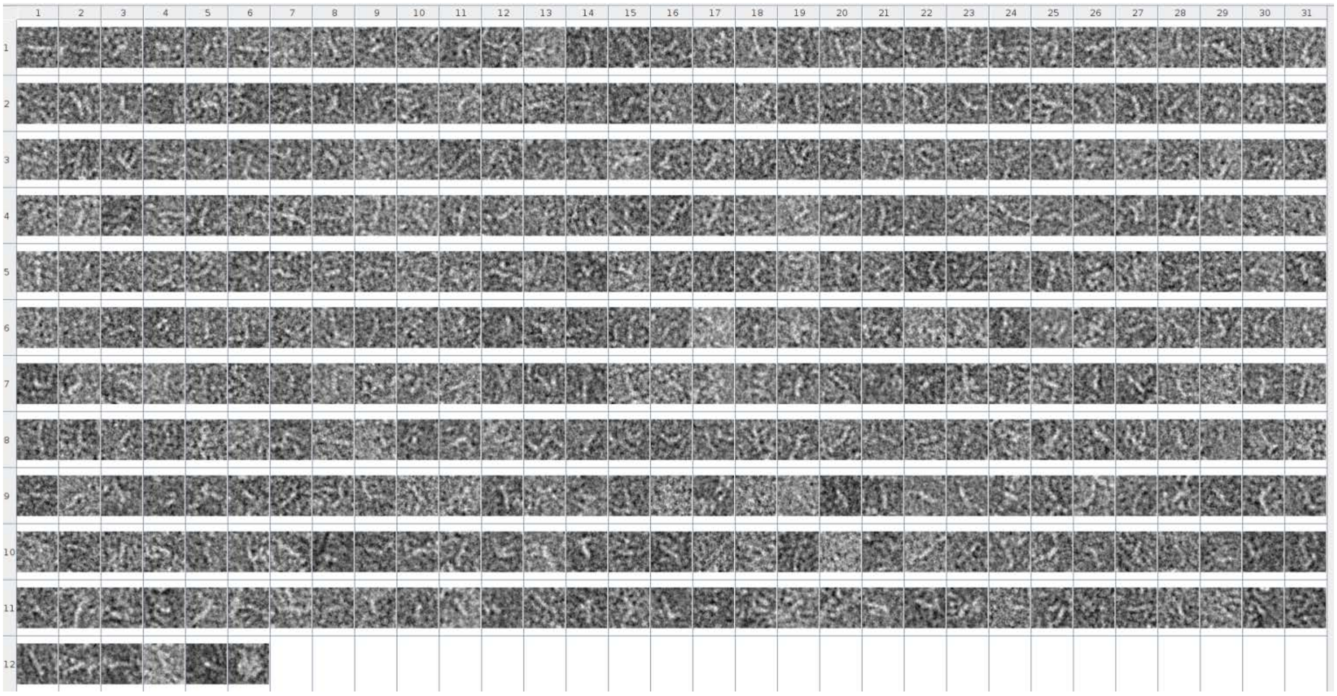

Supplementary Figure 35. TEM images of all 347 particles picked for the POC triplex assembly (**POC1**+**POC2**+**POC3**) in concentrations of 3:2:2 μM.

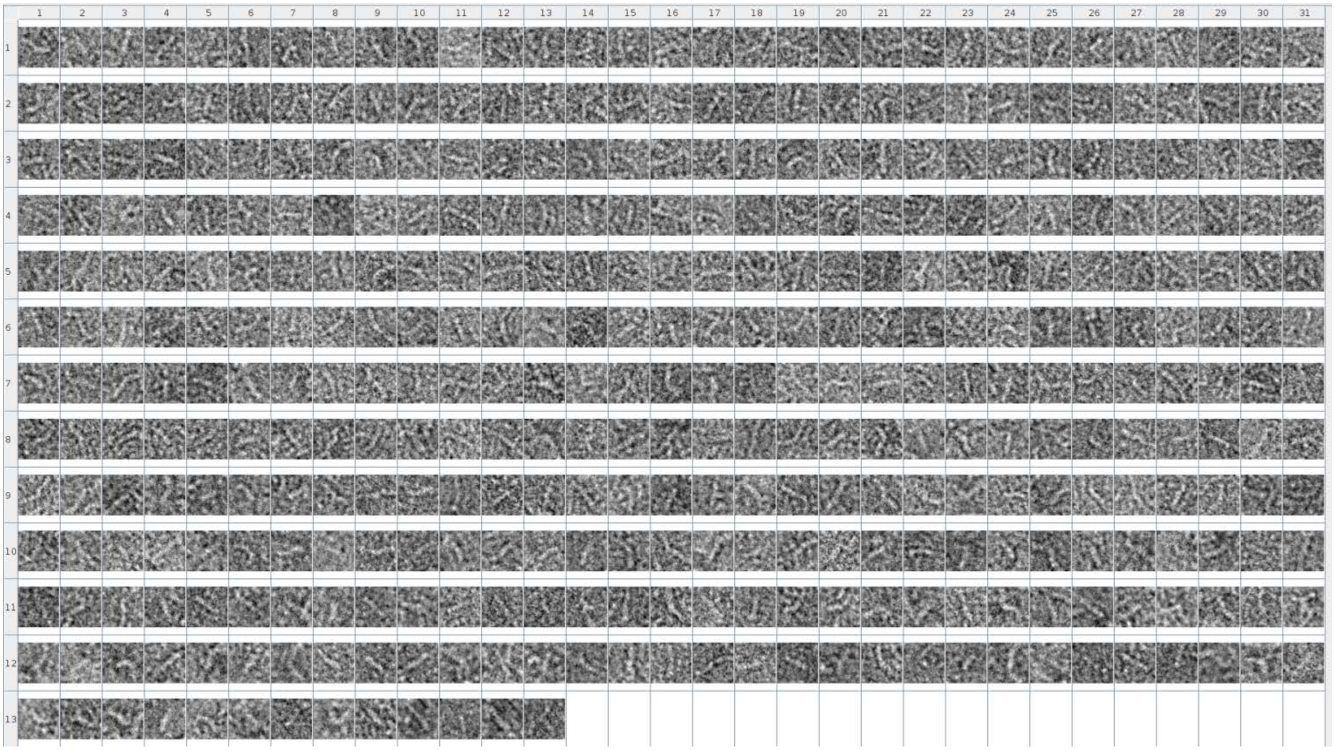

Supplementary Figure 36. TEM images of all 385 particles picked for the POC triplex assembly (**POC1**+**POC2**+**POC3**) in concentrations of 25:25:25 μM.

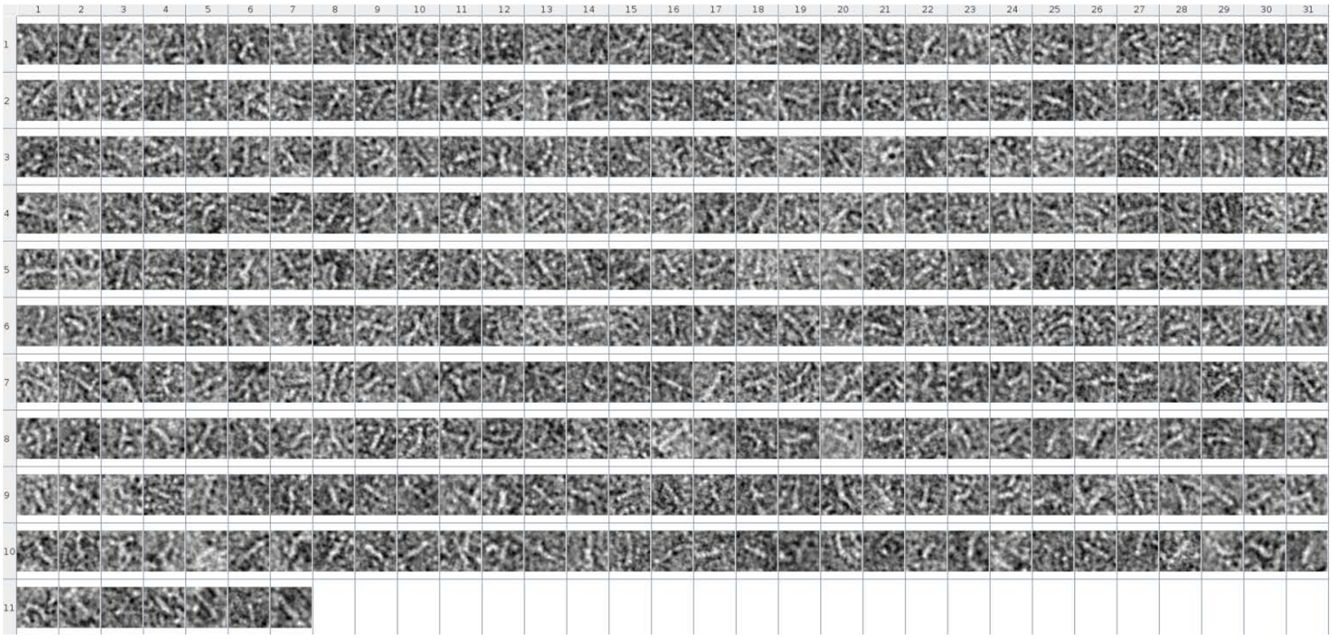

Supplementary Figure 37. TEM images of all 317 particles divided into the straight subclass for the 25:25:25 (25 μM in each POC) concentration.

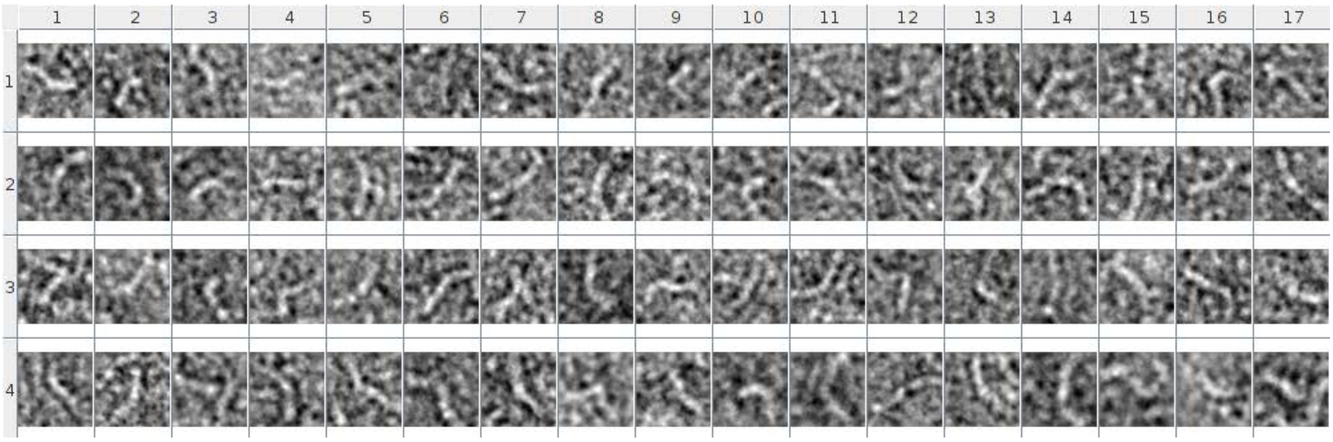

Supplementary Figure 38. TEM images of all 68 particles divided into the bend subclass for the 25:25:25 (25 μM in each POC) concentration.

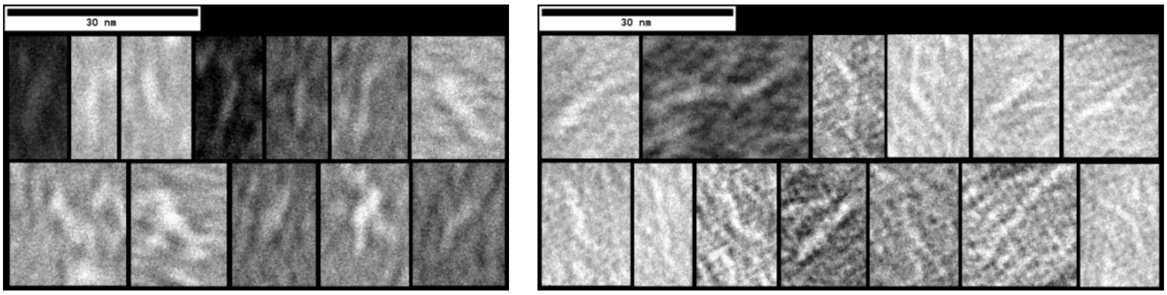

3:2:2 **25:25:25**

Supplementary Figure 39. Close up TEM images showing the different morphology of **POC1**+**POC2**+**POC3** at low "3:2:2" (3 μM in **POC1** (TFO) and 2 μM in **POC2** and **POC3**) and high "25:25:25" (25 μM in each POC).

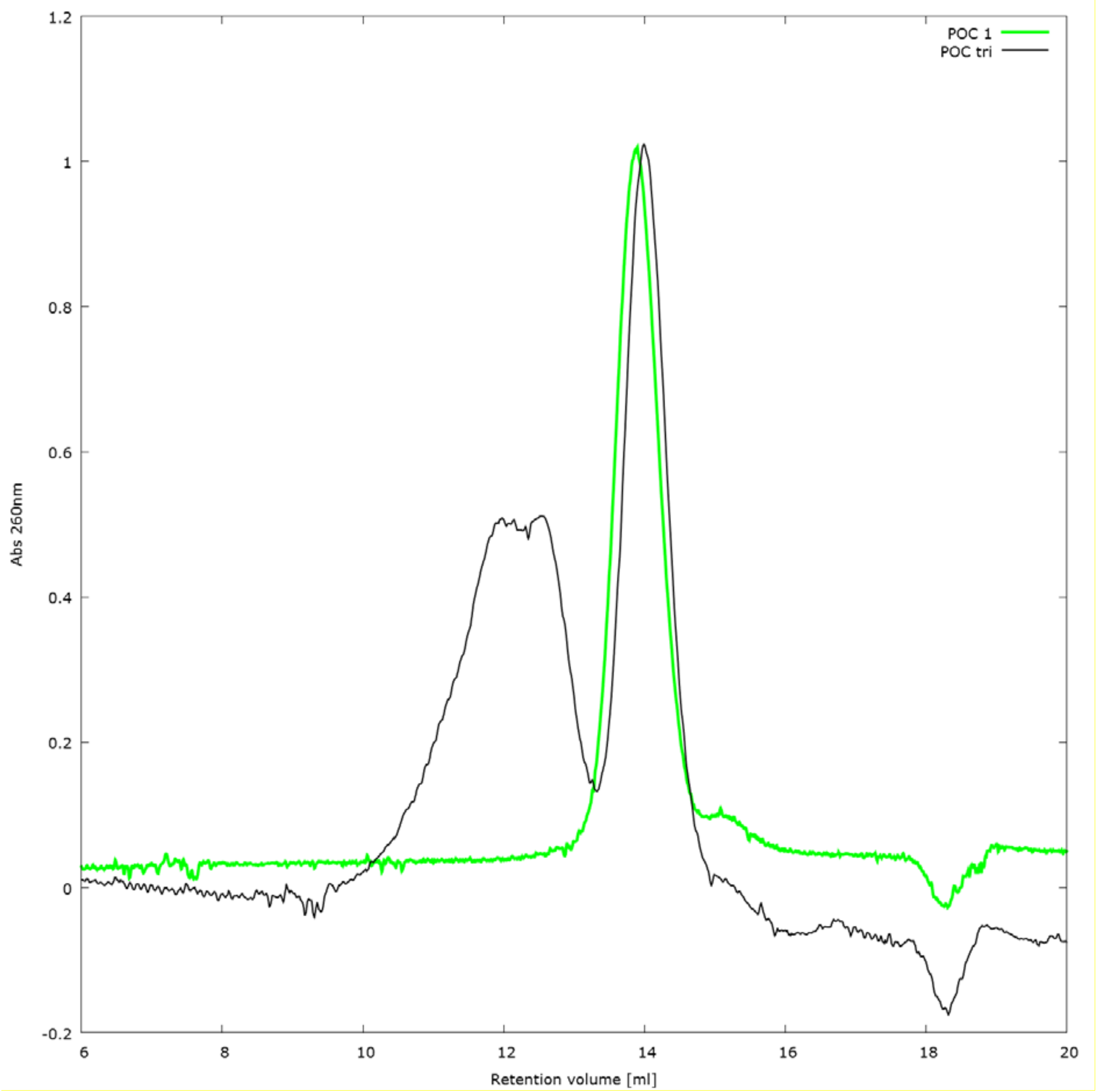

Supplementary Figure 40. SEC: Overlay of the size exclusion chromatograms for **POC1**+**POC2**+**POC3** trimer (POC tri) with the chromatogram for **POC1**. The peak at 14 mL in the **POC1**+**POC2**+**POC3** chromatograms elutes at the same volume as **POC1**. As shown by SAXS, the size of **POC1+POC2+POC3** trimer is very close to that of **POC1** trimer. Here we interpretate that under these SEC conditions the **POC1+POC2+POC3** trimer may partially dissociate from the corresponding dimer of trimers. This could be due to dilution during elution from the column or due to interaction with the column. Re-injection of the peak isolated at 12 mL gave exactly the same size exclusion chromatogram as for POC tri with peaks at 12 mL and 14 mL.

Supplementary Tables

Supplementary Table 1. Mean residue ellipticity of **POC1+POC2+POC3** at 222 nm before and after subtracting the signal of unconjugated oligonucleotides at the same concentration.

**Conc. of each POC (μM)**	**$-[\theta]_{MRE}$ ($deg \cdot cm^2 \cdot dmol^{-1}$)**	**$-[\theta]_{MRE}$ – ONs signal ($deg \cdot cm^2 \cdot dmol^{-1}$)**
0.2	23765	21400
1	35640	32245
4	48645	42745
9	49910	44240
10	51510	46280
11	51940	46495
12	49300	47105
13	53085	49445
14	52995	50475
15	55330	49720
25	52125	46920
37	55335	50650
50	57425	52290

Supplementary Discussion

Further discussion on synthesis of POCs

Initially, Cu(I) catalysed azide-alkyne cycloaddition (CuAAC) chemistry was attempted for synthesis of the needed (POC) units (Supplementary Figure 1)[1-3]. The azido-labelled peptide was synthesized on an automated peptide synthesizer using Fmoc chemistry and a 6-azido hexanoic acid building block for introducing the azido group at the N-terminal. The resulting peptide was purified by RP-HPLC and analyzed by LC-MS and MALDI-TOF MS to confirm purity >95% and constitution (Supplementary Figure 2). All three ONs to be coupled to the peptide fragment were labelled with a terminal alkyne either on the 5’-end or 3’-end (**ON1-H**, **ON2-H** and **ON3-t**[1], Supplemental Figure 1). A number of LNA and 5-methyl-2’-deoxycytidine nucleotide monomers were introduced into the TFO sequence to improve triple helix stability at neutral conditions[4,5]. Attempts at Cu(I) catalysed azide-alkyne couplings were unsuccessful as no desired triazole-linked product was identified in any of the reactions performed using different catalysts[6-9], solvent systems, reaction times and temperatures. Also reactions employing microwave heating were unsuccessful[10]. Next, Cu(I) free ring-strain promoted azide-alkyne couplings was explored for synthesis of the POCs[3,11]. Among various known strained cyclooctynes[12], bicyclo[6.1.0]nonyne (BCN) (Figure 1b) was chosen due to its high second-order rate constant and the fact that it is commercially available as a phosphoramidite monomer ready for coupling using an automated DNA synthesizer[13,14].

ON synthesis was carried out on an automated DNA synthesizer using standard or inverted (3’→5’ or 5’→3’, respectively) solid-phase phosphoramidite chemistry, where the BCN phosphoramidite monomer was attached either on the 5’- or 3’-end of ONs in the last coupling cycle of the synthesis using a manual coupling process[15]. The resulting three ONs were purified by reversed-phase HPLC and their composition and purity (> 95%) confirmed by MALDI-TOF MS and ion-exchange HPLC analysis, respectively (Supplementary Figure 3). The three ONs were desalted either by precipitation (**ON1-BCN**) or using NAP-10 sephadex columns (**ON2-BCN** and **ON3-BCN**, Fig. 1b).

Initial attempts at conjugation by ring-strain promoted reactions were performed in DMSO/H_2O (1:1) for 48 h at both room temperature and 80 °C. However, no detectable coupling products were observed by IE-HPLC or MALDI-TOF MS. We speculate that the

poor reactivity may be caused by the size of the long peptide chain (30-mer) potentially shielding the azido functionality, as well as by the relatively low concentrations of the azido-labelled peptide and the BCN-containing ONs (3.3-5.6 μM of ON, ON:peptide = 1:1.25 equivalent). Gratifyingly, the coupling between **ON1-BCN** and **azidopeptide** was half-completed by microwave-heating at 60 °C after 2 h reaction time (Figure 1c and Supplementary Figure 4A). The product (**POC1**, 30%) was subsequently isolated by IE-HPLC and its composition confirmed by ESI-MS (Supplementary Figure 5). In addition, a notable amount of unreacted **ON1-BCN** was recovered during IE-HPLC purification (92 nmol, 26%).

The same protocol was applied to obtain **POC2** starting from **ON2-BCN** and the **azidopeptide**. After the reaction and removal of all solvents *in vacuo*, only **ON2-BCN** (in limited ~30% yield) was identified when the residue was washed with Milli-Q water (Supplementary Figure 4B, water phase). Intriguingly, despite extensive washings with Milli-Q water a considerable amount of an unknown insoluble white material remained as a fine layer in the round bottled flask. We hypothesized that this unknown substance could be the desired **POC2** product, and by comparison with the **POC1** synthesis we identified the small amounts of inorganic salts (sodium acetate and sodium perchlorate) possibly introduced into **ON1-BCN** during the precipitation process as a putative key difference. In fact, upon addition of Buffer A (0.025 M Tris-HCl, 0.01 M sodium perchlorate, pH 7.6; for IE-HPLC) the unknown white substance mentioned above was dissolved leading to the emergence of a major novel peak by analytical IE-HPLC (Supplementary Figure 4B, buffer phase). And after its isolation a product was isolated in 46% yield with a mass consistent with the calculated molecular weight of **POC2** (Supplementary Figure 5). The **ON2-BCN** obtained from the water phase could be recycled for another round of the conjugation reaction. The same method was successfully applied to the preparation of **POC3** (Supplementary Figure 4C and Supplementary Figure 5) which was isolated in an overall yield of 40%. It is noteworthy that significant secondary structure formation for crude **POC3** was observed by analytical IE-HPLC (Supplemental Figure 4C, buffer phase) as testified by multiple peaks resolving into one peak upon heating to 90 °C followed by slow cooling to room temperature prior to IE-HPLC analysis (Supplementary Figure 4C, bottom).

Efficient and high-yielding preparation of POCs containing longer peptide fragments (⩾ 30-

mers) or proteins is challenging and only very few successful examples have been published via chemical synthesis[16-18]. The cyclic preparation protocol developed here has since been used in our laboratory to synthesize various POCs in which the peptide units are longer than the ON units in terms of number of amino acid relative to nucleotide monomers. The developed protocol therefore serves as a general method for preparation of such conjugates utilizing only 1.25 equivalents of azido-labelled peptides relative to BCN-functionalized ONs, and allowing convenient separation of the desired conjugated products from starting ONs by taking advantage of their different solubility in Milli-Q water. It should be underlined that desalting on NAP column is preferable for BCN-bearing ONs after RP-HPLC purification to prepare them for the above coupling reactions, as this allows control of salt concentration and thus convenient separation between unreacted and conjugated ONs (Supplementary Figure 6).

Further discussion on CD spectroscopy

In Supplementary Figure 18, ON duplex **ON2**+**ON3** was titrated with the triplex-forming oligonucleotide **ON1**. The spectrum of the duplex was characterized by a positive maximum around 275 nm, a negative peak around 240 nm and another small positive maximum around 220 nm. Upon triplex formation, significant spectral differences were induced. While the negative peak at 240 nm was maintained, the positive maximum at 275 nm was shifted to 284 nm. Furthermore, the positive maximum at 220 nm was replaced by a negative signal around 212 nm.

As shown in Supplementary Figure 20 and Supplementary Table 2, the helical content was assessed based on the mean residue ellipticity at 222 nm according to formula 1 commonly used in literature[19], where k is a wavelength dependent factor (2.57 at 222 nm), $[\theta]^{\infty}$ is the molar ellipticity for an infinite helix (-39500 deg·cm^2·dmol^{-1} at 222 nm), and n = number of residues.

$$\% \text{ helical content} = [\theta]/([\theta]^{\infty}\cdot(1 - k/n)) \quad (1)$$

With increasing temperature **azidopeptide** seems to reach a plateau with $[\theta]_{MR}$ at 222 nm around 31000 deg·cm^2·dmol^{-1}, while triplex **POC1+POC2+POC3** continues showing a positive trend to reach values over 50000 deg·cm^2·dmol^{-1}. According to formula 1, these values correspond to 86% and 138% helical content, respectively. This peculiar observation

could be due to the situation that it is not reasonable to treat the triplex **POC1+POC2+POC3** system with the simple model for the assessment the helicity of short peptides. The crystal structure of the parent trimeric coilV_aL_d peptide (PDB ID 1COI) has been determined[20] and a DSSP assignment[21] of secondary structure reveals that 25 of the 30 residues are in a helical stretch corresponding to 86% helicity. This is on the order found for the peptide in solution in this study. Thus, the case in hand shows that the simple models for prediction of helicity based on CD at 222 nm likely overestimate the helicity to a significant degree. In fact, some cases have been reported where the helical content also exceed 100% when calculated using these simple models[22,23]. It has been demonstrated that, at least in particular cases, literature equations that relate helical content to $[\theta]_{222\ nm}$ underestimate both $[\theta]_\infty$ and its length dependence. Consequently, helical content values can be overestimated by 25-50% according to these authors[23]. In this scenario, the larger negative amplitude of the CD is assumed to be caused by a simple increase in the helical folding of the peptide part of **POC1+POC2+POC3**, which can be reasonable as 1) there is only one "loose" terminal of the peptide chain and 2) the kinetics of strand exchange might be significantly slower in **POC1+POC2+POC3** vs the free **azidopeptide**, due to the oligonucleotide hybridization. Both effects would serve to increase the amount of helically folded species at any given point in time for the ensemble. An alternative explanation for the strong negative CD would be if the oligonucleotide signal in the 200-240 nm range is altered in **POC1+POC2+POC3** compared to **ON1+ON2+ON3**, yet the ON triplex titration shown in Supplementary Figure 18 does not lend credit to this option. Finally, an alternative cause of the discrepancy could be that **POC1+POC2+POC3** exhibits a truly altered CD signal due to the helicity of the supramolecular assembly. The relatively strong CD signal of an alpha-helix arises due to the coupling of the helically arrangement of transition dipoles of the peptide groups. We could speculate here, that since the coiled-coil peptide is extended into the oligonucleotide helix there might be a synergistic enhancement of the resulting CD signal, but we do not have any grounds to justify this notion.

Temperature denaturation studies were carried out by temperature scans of CD in the range 20-90 ^oC (Supplementary Figure 21). The unfolding of the coiled coil peptide leads to the disappearance of the α-helix signal together with a decrease of the $\theta_{222nm}/\theta_{208nm}$ ratio. The melting of the ON leads to several spectral changes (Supplementary Figure 21). The transition from the triple helix to the duplex leads to an increase of the positive signal between 305 and 251 nm, together with a small shift of the maximum to lower wavelength.

Furthermore, the wide negative signal between 251 and 206 nm was replaced by a negative signal with a minimum at 240 nm and a positive signal with a maximum at 220 nm. The unfolding of the duplex leads to a decrease in the intensity of the positive signal between 305 and 251 nm and in the negative signal at 240 nm. Temperature series were measured at the selected wavelengths 208, 220, 275 and 285 nm to track both the peptide α-helix and the ON signals (Supplementary Figure 22 and Supplementary Figure 23).

The temperature series at 208 and 220 nm revealed for **ON1+ON2+ON3** a transition from triple helix to duplex with a T_m around 40 ^oC. This transition was observed at around 62 ^oC for **POC1+POC2+POC3**. The unfolding of the peptide was clearly visible with a T_m around 72 ^oC for **azidopeptide** and around 71 ^oC for **POC1+POC2+POC3**. The temperature series at 275 and 285 nm displayed the triple helix and duplex ON transitions. **ON1+ON2+ON3** showed a triple helix and duplex transition with T_m at around 40 ^oC and 62 ^oC, respectively. In the case of **POC1+POC2+POC3**, it was observed that both transitions have higher T_m than those for **ON1+ON2+ON3**, with values around 61 ^oC for triple helix transition and 72 ^oC for duplex transition. Despite the low signal to noise ratio of some transitions, the results showed that there is a thermal stabilization of the duplex and triple helix of **POC1+POC2+POC3** in comparison with the unconjugated **ON1+ON2+ON3**. The T_m values observed by CD spectroscopy are in agreement with those obtained in the UV melting studies.

While the SAXS measurements were performed on samples with concentration of 50 or 150 μM, the melting curves for **ON1+ON2+ON3** and **POC1+POC2+POC3** were measured at 1.0 or 2.0 μM. The CD data shows that neither peptide alone nor the **POC1+POC2+POC3** triple helix was fully folded at concentrations below ~ 12 μM of each strand.

Further discussion molecular modeling

As shown in Supplementary Figure 27, several of the structural snapshots from the MD simulation of the **POC1+POC2+POC3** trimer gave a good approximation of the SAXS data measured at low concentrations (3.6 and 7.2 μM). In the figure, 20 fits to the best MD structures are included, and the predicted curves are barely discernible from each other, reflecting the similarity of the molecular structures. The structure shown as inset in Figure

27a is representative of all the 20 best MD structures, and reveals a kink in the linker region, imparting a characteristic “L-shape” to the overall **POC1+POC2+POC3** trimer structure.

Analysis of the ensemble of **POC1+POC2+POC3** dimers of trimers generated by molecular modeling and selection by agreement with SAXS at high concentration (50 µM) gave optimal results for the structure shown in Supplementary Figure 29 (Table 2b), with $\chi = 11$ for the fit between calculated and experimental scattering curves (Supplementary Figure 28A and 28B). This dimer of trimers was built from the **POC1+POC2+POC3** trimer extracted after 13.3 ns of MD simulation, where the linker had rearranged substantially yielding the overall L-shape discussed above for the best single **POC1+POC2+POC3** trimer model. Importantly, a particularly good fit was noted in the region 0.01 - 0.2 $Å^{-1}$ where the experimental data are most robust. The discrepancy between the calculated and experimental SAXS curves after ~0.25 $Å^{-1}$, reflects the neglect of detailed modeling of solvation, ions and possibly the fine structure of the POC model.

Robustness of the computational approach. In order to probe the robustness of the combined molecular modeling/SAXS approach, we calculated SAXS scattering curves for the 750 molecular dynamics structure snapshots of the **POC1**+**POC2**+**POC3** trimer and performed fitting against the experimental SAXS curve for **POC1**+**POC2**+**POC3** at high concentration (50 µM), i.e. where **POC1**+**POC2**+**POC3** is expected to form dimers of trimers. The results (Supplementary Figure 28C) clearly indicated the inability of any of the **POC1**+**POC2**+**POC3** trimer structures to fit the 50 µM SAXS data.

Further discussion on TEM imaging

The POCs were very difficult to image on the TEM by negative stain as the structure resolution was limited by the stain technique and small size. A more detailed structural analysis from EM would require to-go into cryo EM as the salt grain size is about the 20 Å which corresponds to the width of the conjugates.

Additionally, higher order aggregates were observable in many areas which we suspect not to be present in bulk solution but rather as an artefact of the TEM grid preparation. Also the carbon layer on top of the grid often absorbs molecules increasing the local concentration at

the surface compared to the bulk. From the raw images only individually distinguishable particles was analyzed (Supplementary Fig. 33) where the length and width was measured and aspect ratio calculated as seen in Figure 4. In particular Supplementary Figure 34a with the 3:2:2 μM concentration of POCs show a high level of aggregates on the carbon surface, but between these individual conjugates are visible. Supplementary Figure 34b on the other hand is very densely crowed with POCs, as expected due to the very high concentration, which again makes it difficult to analyze, but conjugates are again distinguishable and have in general a more elongated shape with a higher aspect ratio. Also this high density is the reason for a concentration of 25 μM instead of 50 μM during this experiment but this dilution of the sample was done seconds before incubation on the TEM grid. Furthermore, artifacts can arise from the drying process as the shell of hydration is lost which can cause the conjugate to change shape and stable configuration.

The raw images were processed by the Scipion software to create class average images of selected particles (Supplementary Figure 33). A total of 347 and 385 particles were selected for the 3:2:2 (Supplementary Figure 35) and 25:25:25 (Supplementary Figure 36) concentrations, respectively, and each grouped into bended and straight classes. Unfortunately fully automatic picking was not possible due to the high concentration hence only manual and partly supervised particle picking was possible. Due to the flexibility of the particles, especially the 25:25:25 concentration, we divided the particles into straight (Supplementary Figure 37) and bended (Supplementary Figure 38) shapes for averaging. The bended class in Figure 4 is dominated by highly bended particles, whereas the straight class contained straight to lightly bended particles, which explains the shorter appearance and end fuzziness. Examples of raw close up image POCs is shown in Supplementary Figure 39.

Supplementary Methods

All basic chemical reagents were purchased from Sigma-Aldrich or Fluka and used without purification. DNA phosphoramidite monomers (standard and inverted), solid supports and additional reagents were purchased from Sigma-Aldrich, GE Healthcare or Glen Research. LNA-T, 5'-methyl-dC and cyclooctyne (BCN CEP II) phosphoramidite monomers were purchased from Exiqon, Link technologies and Berry&Associates, respectively. All amino acids, HBTU, HOBt, were purchased from GL Biochem (Shanghai) Ltd. DIPEA, piperidine and TFA were purchased from Iris Biotech GmbH. TES was purchased from Sigma-Aldrich (Denmark) and TentaGel S RAM resins were purchased from Rapp Polymere. For all experiments, concentrations of POCs and ONs were determined using theoretical values for ε_{260nm} of $1.11 \cdot 10^5$ $M^{-1} \cdot cm^{-1}$, $2.41 \cdot 10^5$ $M^{-1} \cdot cm^{-1}$ and $2.01 \cdot 10^5$ $M^{-1} \cdot cm^{-1}$ for **POC1**/**ON1**, **POC2**/**ON2** and **POC3**/**ON3**, respectively. A theoretical ε_{280nm} of $1.49 \cdot 10^3$ $M^{-1} \cdot cm^{-1}$ was used to establish the concentration of **azidopeptide**.

Peptide synthesis, purification and analysis

The synthesis was carried out using an automated peptide synthesizer (Biotage SYRO II). Analytical HPLC was performed on a Dionex Ultimate 3000 system with Phenomenex Germini C18 column (3μm, 50 × 4.6 mm) and a linear gradient flow of CH_3CN-H_2O (0.1% formic acid), column oven thermostated to 42 °C, connected to an ESI-MS (MSQ Plus Mass Spectrometer, Dionex). Purification of the peptide was performed on a preparative Dionex Ultimate 3000 HPLC with a C18 column from FeF chemicals (Denmark) (5 µm, 250 × 21.2 mm, 300Å). CH_3CN-H_2O (0.1% TFA) was used as eluent with a flow of 15 ml/min. High-resolution mass spectrometry (HR-MS) was performed on a SolariX XR FT-ICR-MS instrument from Bruker by direct injection with ionization in positive electrospray mode (Supplemental Figure 2). The peptide was synthesized using Tentagel S RAM (4 × 2 g; 4 × 0.5 mmol; loading 0.25 mmol/g) resins as solid support that initially was swelled in CH_2Cl_2. Coupling of the amino acids (5 eq) were utilized via standard Fmoc solid phase peptide synthesis using Oxyma (5 eq) and DIC (5 eq) in DMF as coupling reagents. Coupling time for 2 × 2 h. Fmoc deprotection was carried out using piperidine-DMF (2:3) for 3 min followed by piperidine-DMF (1:4) for 15 min. The peptide was released and deprotected by treatment with 95:2.5:2.5 TFA:TES:H_2O for 2 h. After removing TFA under a flow of nitrogen atmosphere, the peptide was precipitated with cold diethylether giving a white

powder. The crude product was purified by preparative RP-HPLC (gradient 5-65% over 25 min) and then analyzed by HR-ESI mass spectrometry: calculated for $C_{155}H_{258}N_{42}O_{48}$ $[M+4H]^+$: (869.98325); found: 869.98657 (3.3 ppm).

Oligonucleotide synthesis, purification and analysis

Oligonucleotide synthesis was carried out on a PerSeptive Biosystems Expedite 8909 automated DNA/RNA synthesizer or an ÄKTA oligopilot plus 10 system in 1.0 μmol scale (CPG support) using the phosphoramidite approach and following manufacturer's standard protocols. The coupling time for standard, inverted, 5-methyl-dC and LNA monomers was 144 s and stepwise coupling efficiencies in all cases were >98.0%, which were determined by the absorbance of the DMT cation at 495 nm. Bicyclo[6.1.0]nonyne (BCN) (BCN CEP II) was incorporated into oligonucleotides either on the 3'-end or the 5'-end via manual-coupling using 5-[3,5-bis(trifluoromethyl)phenyl]-*H*-tetrazole (0.25 M, in anhydrous acetonitrile) as activator and extended coupling time (15 min). 3'-Incorporation of BCN CEP II was accomplished through inverted oligonucleotide synthesis (5'→3') using inverted DNA phosphoramidite monomers on a universal solid support (Universal 40, GE Healthcare). For oligonucleotides with cycloocytne addition on the 5'-end, cleavage from solid support and removal of nucleobase protecting groups were performed using 28% aqueous ammonia for 16 h at 55 °C, whereas oligonucleotides synthesised on a universal solid support were cleaved from the support using 3.5 M ammonia in methanol (1 mL, 2 h, at room temperature) and deprotected at 55 °C for 16 h after addition of 30% aqueous ammonia (1 mL) into the cleavage mixture. The resulting oligonucleotides were purified by RP-HPLC using a Waters System 600 HPLC equipment equipped with a Waters XBridge BEH C18-column (5 μm, 100 mm × 19 mm). Elution was performed starting with an isocratic hold of A-buffer for 5 min followed by a linear gradient to 70% B-buffer over 16.5 min at a flow rate of 5.0 mL/min (A-buffer: 0.05 M triethylammonium acetate in Milli-Q water, pH 7.4; B-buffer: 25% A-buffer, 75% acetonitrile). After removal of the solvents under a flow of nitrogen, the oligonucleotide products were desalted either using NAP-10 Sephadex columns (GE Healthcare) according to the manufacturer's instructions (**ON2-BCN** and **ON3-BCN**) or by precipitation in acetone (**ON1-3**, **ON1-BCN**). Mass spectra of oligonucleotides were recorded on a Bruker Daltonics Microflex LT MAIDI-TOF MS instrument in ES^+ mode (representative MS in Supplemental Figure 3). Analytical IE-HPLC traces were recorded on a Merck-Hitachi Lachrom system

equipped with a DNAPac PA100 analytical column (13 µm, 250 mm × 4 mm) heated to 60 °C. Elution was performed with an isocratic hold of buffer B (10%), starting from 2 min hold using 2% Buffer A in Milli-Q water, followed by a linear gradient to 30% buffer A in 23 min at a flow rate of 1.1 mL/min (buffer A: 1.0 M sodium perchlorate; buffer B: 0.25 M Tris-Cl, pH 8.0) (representative IE-HPLC traces in Supplemental Figure 3). Concentrations of purified oligonucleotides were determined by UV absorption measurements at 260 nm.

UV spectra of TFO, duplexes and triplexes

Representative UV absorption curves of **ON1** and unmodified duplexe **ON2**+**ON3** are shown in Supplemental Figure 7. For easy comparison, both duplex and triplex melting curves were recorded at 275 nm. UV spectra were recorded of all samples before carrying out a UV melting study (Supplemental Figure 24).

Ultraviolet duplex melting studies

To determine duplex melting temperatures (T_m values), UV melting studies were carried out on a Perkin Elmer Lambda 35 UV/Vis Spectrometer using Hellma SUPRASIL synthetic quartz 10 mm path length cuvettes, monitoring at 275 nm at a concentration of 1.0 µM of each strand and a volume of 1.0 mL. Samples were prepared as follows: The oligonucleotide (or the POC) and their corresponding complementary strand (or POC) were mixed in 1:1 ratio in 2 mL Eppendorf tubes before medium salt buffer (2 times, 11.7 mM sodium phosphate, 200 mM NaCl, 0.20 mM EDTA, pH 7.0, 500 µL) was added followed by addition of Milli-Q water to a total volume of 1.0 mL. The samples were denatured by heating to 90 ˚C in a water-bath followed by slow cooling to room temperature before they were transferred into the cuvettes. The UV absorbance at 275 nm as a function of time was recorded while the temperature was increased linearly from 10 °C to 80 °C at a rate of 1.0 °C/min programmed by a Peltier temperature controller. Three separate melting curves were measured and T_m values were calculated using UV-WinLab software, taking an average of the three T_m values calculated. In the same way, annealing temperature (T_a) was also calculated from three annealing curves. Standard deviations on T_m and T_a values were calculated using the confidence intervals derivable with Student’s t distribution of 95% confidence and the appropriate degrees of freedom.

Ultraviolet triplex melting studies

To determine triplex melting temperatures (T_m), UV melting studies at 275 nm were carried out on a Perkin Elmer Lambda 35 UV/Vis Spectrometer using Hellma SUPRASIL synthetic quartz 10 mm path length cuvettes, with a concentration of 1.0 µM for each of the two duplex-forming strands, a concentration of 1.5 µM for the triplex-forming strand, and a total volume of 1.0 mL. Samples were prepared as follows: The triplex-forming strand (**ON1** or **POC1**) and the duplex were mixed in a 2 mL Eppendorf tube followed by addition of medium salt buffer (2 times, 11.7 mM sodium phosphate, 200 mM NaCl, 0.20 mM EDTA, pH 7.0 or 7.5, 500 µL), to which Milli-Q water was added to a total volume of 1.0 mL. Thus, all triplex samples were dissolved in 1 × buffer condition (5.8 mM sodium phosphate, 100 mM NaCl and 0.10 mM EDTA). The samples were denatured by heating to 90 ˚C in a water-bath followed by slow cooling to room temperature and storage overnight in a fridge before they were transferred into cuvettes. The UV absorbance at 275 nm as a function of time was recorded while the temperature was increased linearly from 10 °C to 80 °C at a rate of 0.5 °C/min programmed by a Peltier temperature controller. Three separate melting curves were measured and T_{m} values were calculated using UV-WinLab software, taking an average of the three T_{m} values calculated. In the same way, annealing temperature (T_{a}) was also calculated from three annealing curves. (Supplemental Figures 9, 10, 11, 12 and 13). Standard deviations on T_{m} and T_{a} values were calculated using the confidence intervals derivable with Student's t distribution of 95% confidence and the appropriate degrees of freedom.

Non-denaturing gel analysis

The same concentrations of strands as in melting experiments (see above) were used in medium salt buffer (5.8 mM sodium phosphate, 100 mM NaCl and 0.10 mM EDTA). In total six samples were prepared, i.e. oligonucleotide duplex (**ON2+ON3**), POC duplex (**POC2+POC3**), oligonucleotide triplex (**ON1+ON2+ON3**), POC triplex (**POC1+POC2+POC3**) and two duplex hybrids (**ON2+POC3** and **POC2+ON3**). All samples were denatured by heating to 90 ˚C in a water-bath followed by slow cooling to room temperature and storage overnight in a fridge before they [5 µL per sample under pH 8.3 at room temperature and 4 ˚C; 5 µL per sample under pH 7.0 at room temperature; 10 µL per sample under pH 7.0 at 4 ˚C; each was mixed with equivalent volume of Ficoll solution

(5 µL or 10 µL, 15%, w/w in 0.5× TBE buffer)] were loaded together with O'GeneRuler Ultra Low Range DNA Ladder onto a 13% non-denaturing polyacrylamide gel for electrophoresis at a low voltage (250 V) and visualisation by UV excitation at 260 nm on a Syngene Geni Imager after ethidium bromide staining.

Circular dichroism (CD) spectroscopy

Far UV CD data were recorded on a JASCO J-815 calibrated with ammonium d-10-camphorsulfonate and the data acquired using 0.01, 0.1, 0.2, 0.5 or 1 cm path-length cells from Hellma. The solutions were prepared in 5.83 mM phosphate buffer pH 7.0 with 100 mM NaCl and 0.1 mM EDTA. CD temperature scans and series were performed between 20 and 90 ^{o}C in a thermostated cell holder in the CD instrument. CD scans were measured at + 2 ^{o}C intervals, while + 1 ^{o}C intervals were used for the temperature series. T_m values based on CD results were determined as maximum/minimum of the first derivative of the temperature series (see Supplementary Figure 23)

Small angle X-ray scattering (SAXS)

SAXS data were obtained at beamline B21 at the Diamond light Source, situated in Oxfordshire, Britain. For all experiments, a sample to detector distance of 3.9 m and 12.4 keV X-rays was used. For each measurement about 20 µL of sample was loaded using the temperature controlled sample changer robot at the beamline. The samples were measured at 10 ^{o}C. Measurements required relatively high concentrations, which were 50 µM when the POCs were studied separately; the **azidopeptide** was measured at 150 µM; the triplex ONs (**ON1+ON2+ON3**) was measured at 50 µM for each ON; while the concentration for triplex POCs (**POC1**+**POC2**+**POC3**) was 50 µM for each POC. The 50 µM concentration samples were measured for 10 min while the 150 µM concentration samples were measured for 5 min. SAXS data for the low concentrations of **POC1**+**POC2**+**POC3** were measured at the European Synchrotron Radiation Facility (ESRF). For each SAXS data set, the sample and buffer background were automatically azimuthally averaged. The data were then background subtracted and normalized using the available software at the beamline. This produced data with the scattering intensity, (I), as a function of the scattered momentum transfer, q, defined as $q = 4\pi \sin(\theta) / \lambda$, where 2θ is the scattering angle, and λ is the wavelength of the incoming

radiation. Absolute scale calibration was done using water as standard. Real space representations, in terms of the so-called pair-distance distribution functions, *p(r),* of the SAXS data, were obtained using the software GNOM[24] implemented in the current version of the ATSAS suite[25]. Structural modelling was done using the online version of the program MONSA[26]. Ten structures were averaged and the average structure is presented. I(0) was calculated from the experimental data using $I(0)=n*\Delta\rho^2*V^2$ where I(0) is the scattering intensity at zero angle, n is the concentration, Δρ is the excess scattering length density and V is the volume of the particle. Here a standard volume of 1.35 $Å^3$ per amino acid, 400 $Å^3$ per adenine and thymine nucleotides, 370 $Å^3$ per guanine and cytosine nucleotides, and 30 $Å^3$ for at water molecule was used. Δρ is proportional to the number of electrons per volume from the molecule and solvent, and was calculated using the Thompson electron radius of 2.818 x 10^{-13} cm.

Molecular Modeling

Molecular dynamics simulations of POC1+POC2+POC3

Construction of the model commenced with preparation of the **ON1+ON2+ON3**, linker, and **azidopeptide** components. The **ON1+ON2+ON3** triple helix was built using Accelrys Discovery Studio 4.1[27] using standard nucleotides, followed by manual editing of the base pairs in Maestro[28] to yield the structure corresponding to the **ON1+ON2+ON3** sequences. Local geometry optimization was carried out to relax the edited residues in Maestro with the OPLS_2005 force field[29]. The **azidopeptide**-trimer was prepared from the coil-VaLd crystal structure[20], including addition of the extra *N*-terminal Tyr residues and hydrogens. The linker was constructed with the Maestro build tool, and subjected to conformational search with the mixed MCMM/low-mode search method of Macromodel[30], giving an ensemble of conformations from which extended conformations were selected for further modeling. The **ON1+ON2+ON3**, linkers, and **azidopeptide** were merged to form the **POC1+POC2+POC3** model in Maestro by successive superposition of the structural elements and local geometry optimization with Macromodel using the OPLS_2005 force field. The resulting model was subjected to molecular dynamics (MD) simulations to sample the conformations of the linker and the relative arrangement of the oligopeptide and -nucleotide parts. To this end, the **POC1+POC2+POC3** model was solvated in a cubic box of TI3P waters[31], with 58 charge compensating Na^+ ions and an ionic background of 100 mM NaCl[32], yielding a layer of at least 16 Å TIP3P waters on each side of **POC1+POC2+POC3** and a total system size of

459166 atoms. MD simulations were carried out for 15 ns with the GPU version of Desmond[1] in the NPT ensemble at 300 K, using the OPLS_2005 force field, and saving all atomic coordinates every 20 ps yielding 750 structural snapshots (MD frames). Since the OPLS_2005 force field does not provide adequate parameters for nucleic acids, the atoms of **ON1+ON2+ON3** were restrained to their initial positions during MD simulations, allowing free movement of all other atoms.

POC1+POC2+POC3 SAXS prediction from molecular dynamics ensemble

Since the molecular dimensions obtained from SAXS for the low concentration **POC1+POC2+POC3** samples indicated trimers and no higher oligomers, we used each of the 750 structural snapshots from the MD ensemble as proxy models for the prediction of the SAXS experimental scattering curves at 3.6 and 7.2 µM using FoXS[35] with default settings. The discrepancy between experimental and theoretical SAXS curves as quantified by the FoXS χ-parameter was used to select the 20 best structures from the MD ensemble. The 20 best predicted SAXS curves, and the structure for the single best model, are shown in Supplementary Figure 27.

Higher-order POC1+POC2+POC3 oligomer models without symmetry constraints

At high concentration (50 µM), molecular dimensions from SAXS indicated **POC1+POC2+POC3** dimers of trimers. In order to model dimers of the **POC1+POC2+POC3** trimer, the trimer structure was extracted from every tenth frame of the MD trajectory and rigidly docked against a copy of itself using PatchDock[33] followed by refinement with FireDock[34] yielding a structurally diverse ensemble of dimer of trimers. SAXS scattering curves were calculated for the ensemble of dimer of trimers with FoXS and fitted to the experimental SAXS curve using default settings. Again, the discrepancy between experimental and theoretical SAXS curves as quantified by the FoXS χ-parameter was used to select the dimer of trimer models that were most consistent with the experimental SAXS data, see Supplementary Figures 28, 29 and Table 2b.

Higher-order POC1+POC2+POC3 oligomer models with symmetry constraints

Each of the 750 structural snapshots of **POC1+POC2+POC3** produced by MD simulations (see above) was used as input for SymmDock for building both symmetric dimers and trimers of **POC1+POC2+POC3**. For each MD frame, 100 symmetrical models for both the dimer-of-trimer and trimer-of-dimer were produced, yielding a total of 75000 models (some

identical) for both types of oligomer. SAXS curves were calculated using FoXS for all models, and fitted against the experimental SAXS data in the robust 0.01 to 0.25 $Å^{-1}$ range. FoXS χ-parameters for all dimer-of-trimer (red) and trimer-of-trimer (green) models are plotted in Supplementary Figure 30. Models with low χ were selected for visual inspection in order to exclude structures with implausible features (e.g. crossing linkers) sometimes produced by docking. An example of a low-χ symmetrical trimeric **POC1+POC2+POC3** model with calculated SAXS curve is shown in Supplementary Figure 31.

Multistate Modeling of SAXS profiles

MultiFoXS was used to fit multistate models consisting of dimers of trimers and trimers of trimers **POC1+POC2+POC3** to the experimental SAXS curve. To this end, a plausible symmetrical trimer-of-trimer molecular model (i.e. with low χ and physically reasonable upon visual inspection) was selected (Supplementary Figure 31**)** and used as input to MultiFoXS together with the ensemble of 750 molecular dynamics snapshots of the **POC1+POC2+POC3** model, yielding 750 calculated composite SAXS curves. The lowest FoXS χ-value for the fit against the experimental SAXS curve in the range 0.01 to 0.25 $Å^{-1}$ range was 14.07, and assumed a linear structure of **POC1+POC2+POC3**. See Supplementary Figure 32. The calculated curve is clearly inferior to that of the dimer of **POC1+POC2+POC3** (Supplementary Figure 28).

Transmission electron microscopy (TEM)

Samples were adsorbed onto glow-discharged carbon-coated Cu200 (20-30nm) TEM grids (TED Pella Inc., Redding, Ca, USA) for 1 min, quickly dried and immediately followed by a negative stain incubation with newly filtered 1% aqueous uranyl acetate solution for 30 seconds and left to dry in air prior to imaging. Imaging was done using a FEI Tecnai G2 Spirit electron microscope operated at 120kV. Acquisition of images was done using a bottom mounted TVIPS CMOS 4k camera (TEM-cam-F416). Imaging magnifications of x67000, x110000, x150000 and x220000 was used. For better contrast and reduced noise, images were captured as a series of 5 or more and averaged by the EM-Menu interface software provided by TVIPS. Particle dimensions and shape were afterwards analyzed with only single distinguishable particles included.

Size-Exclusion Chromatography (SEC)

A Superdex 200 (10/300) (GE Healtcare) column was equilibrated with buffer at isocratic flow of 0.5 mL/min at 4 ^{o}C. The buffer was 5.83 mM sodium phosphate buffer pH 7.0 with 100 mM NaCl and 0.1 mM EDTA. The injection volumes were 20 µL and sample elutions were monitored at 215 nm, 260 nm and 280 nm. Both, **POC1** and **POC1**+**POC2**+**POC3** triplex POC samples were injected at 50 µM. See Supplementary Figure 40 for results.

Supplementary References

1. Astakhova, I. K., Hansen, L. H., Vester, B. & Wengel, J. Peptide-LNA oligonucleotide conjugates. *Org. Biomol. Chem.* **11**, 4240-4249 (2013).
2. El-Sagheer, A. H. & Brown, T. Click chemistry with DNA. *Chem. Soc. Rev.* **39**, 1388-1405 (2010).
3. Tang, W. & Becker, M. L. "Click" reactions: A versatile toolbox for the synthesis of peptide-conjugates. *Chem. Soc. Rev.* **43**, 7013-7039 (2014).
4. Højland, T. *et al.* LNA (locked nucleic acid) and analogs as triplex-forming oligonucleotides. *Org. Biomol. Chem.* **5**, 2375-2379 (2007).
5. Povsic, T. J. & Dervan, P. B. Triple helix formation by oligonucleotides on DNA extended to the physiological pH range. *J. Am. Chem. Soc.* **111**, 3059-3061 (1989).
6. Chan, T. R., Hilgraf, R., Sharpless, K. B. & Fokin, V. V. Polytriazoles as Copper(I)-Stabilizing Ligands in Catalysis. *Org. Lett.* **6**, 2853-2855 (2004).
7. Hong, V., Presolski, S. I., Ma, C. & Finn, M. G. Analysis and optimization of copper-catalyzed azide-alkyne cycloaddition for bioconjugation. *Angew. Chem. Int. ed.* **48**, 9879-9883 (2009).
8. Chan, T. R. & Fokin, V. V. Polymer-supported copper(I) catalysts for the experimentally simplified azide-alkyne cycloaddition. *QSAR Comb. Sci.* **26**, 1274-1279 (2007).
9. Eltepu, L., Jayaraman, M., Rajeev, K. G. & Manoharan, M. An immobilized and reusable Cu(I) catalyst for metal ion-free conjugation of ligands to fully deprotected oligonucleotides through click reaction. *Chem. Commun.* **49**, 184-186 (2013).
10. Barge, A., Tagliapietra, S., Binello, A. & Cravotto, G. Click Chemistry Under Microwave or Ultrasound Irradiation. *Curr. Org. Chem.* **15**, 189-203 (2011).
11. Jewett, J. C. & Bertozzi, C. R. Cu-free click cycloaddition reactions in chemical biology. *Chem. Soc. Rev.* **39**, 1272-1279 (2010).
12. King, M. & Wagner, A. Developments in the field of bioorthogonal bond forming reactions-past and present trends. *Bioconjugate Chem.* **25**, 825-839 (2014).
13. Dommerholt, J. *et al.* Readily accessible bicyclononynes for bioorthogonal labeling and three-dimensional imaging of living cells. *Angew. Chem. Int. ed.* **49**, 9422-9425 (2010).
14. Hodges, J. C., Van Delft, F. L., Van Berkel, S. S., Verkade, J. & Berry, D. A. Preparation of bicyclo[6.1.0]non-4-yne reagents for chemical modification of oligonucleotides. Patent WO2013036748A1 (2013).
15. Rajwanshi, V. K., Hakansson, A. E., Dahl, B. M. & Wengel, J. LNA stereoisomers: xylo-LNA (beta-D-xylo configured locked nucleic acid) and alpha-L-LNA (alpha-L-ribo configured locked nucleic acid). *Chem. Commun.*, 1395-1396 (1999).
16. Humenik, M. & Scheibel, T. Nanomaterial building blocks based on spider silk-oligonucleotide conjugates. *ACS Nano* **8**, 1342-1349 (2014).
17. Lee, M.-k. & Lim, Y.-b. Facile synthesis, optical and conformational characteristics, and efficient intracellular delivery of a peptide-DNA conjugate. *Bioorg. Med. Chem.* **22**, 4204-4209 (2014).
18. Liang, S. I., McFarland, J. M., Rabuka, D. & Gartner, Z. J. A modular approach for assembling aldehyde-tagged proteins on DNA scaffolds. *J. Am. Chem. Soc.* **136**, 10850-10853 (2014).
19. Chen, Y. H., Yang, J. T. & Chau, K. H. Determination of helix and beta-form of proteins in aqueous-solution by circular-dichroism. *Biochemistry* **13**, 3350-3359 (1974).

20. Ogihara, N. L., Weiss, M. S., Degrado, W. F. & Eisenberg, D. The crystal structure of the designed trimeric coiled coil coil-V(a)L(d): Implications for engineering crystals and supramolecular assemblies. *Protein Sci.* **6**, 80-88 (1997).
21. Kabsch, W. & Sander, C. Dictionary of protein secondary structure-pattern-recognition of hydrogen-bonded and geometrical features. *Biopolymers* **22**, 2577-2637 (1983).
22. Lazo, N. D. & Downing, D. T. Circular dichroism of model peptides emulating the amphipathic alpha-helical regions of intermediate filaments. *Biochemistry* **36**, 2559-2565 (1997).
23. Wallimann, P., Kennedy, R. J. & Kemp, D. S. Large circular dichroism ellipticities for N-templated helical polypeptides are inconsistent with currently accepted helicity algorithms. *Angew. Chem. Int. ed.* **38**, 1290-1292 (1999).
24. Svergun, D. I. Determination of the regularization parameter in indirect-transform methods using perceptual criteria. *J. Appl. Crystallogr.* **25**, 495-503 (1992).
25. Petoukhov, M. V. *et al.* New developments in the ATSAS program package for small-angle scattering data analysis. *J. Appl. Crystallogr.* **45**, 342-350 (2012).
26. Svergun, D. I. Restoring low resolution structure of biological macromolecules from solution scattering using simulated annealing. *Biophys. J.* **76**, 2879-2886 (1999).
27. Dassault Systèmes BIOVIA, Discovery Studio Modeling Environment, Release 4.5, San Diego: Dassault Systèmes, 2015.
28. Schrödinger Release 2014-1: Desmond Molecular Dynamics System, version 3.7, D. E. Shaw Research, New York, NY, 2014. Maestro-Desmond Interoperability Tools, version 3.7, Schrödinger, New York, NY, 2014.
29. Banks, J. L. *et al.* Integrated modeling program, applied chemical theory (IMPACT). *J. Comput. Chem.* **26**, 1752-1780 (2005).
30. MacroModel, Schrödinger Release 2013-3: MacroModel, version 10.2, Schrödinger, LLC, New York, NY, 2013.
31. Jorgensen, W. L., Chandrasekhar, J., Madura, J. D., Impey, R. W. & Klein, M. L. Comparison of simple potential functions for simulating liquid water. *J. Chem. Phys.* **79**, 926-935 (1983).
32. Jensen, K. P. & Jorgensen, W. L. Halide, ammonium, and alkali metal ion parameters for modeling aqueous solutions. *J. Chem. Theory Comput.* **2**, 1499-1509 (2006).
33. Schneidman-Duhovny, D., Inbar, Y., Nussinov, R. & Wolfson, H. J. PatchDock and SymmDock: servers for rigid and symmetric docking. *Nucleic Acids Res.* **33**, W363-W367 (2005).
34. Mashiach, E., Schneidman-Duhovny, D., Andrusier, N., Nussinov, R. & Wolfson, H. J. FireDock: a web server for fast interaction refinement in molecular docking. *Nucleic Acids Res.* **36**, W229-W232 (2008).
35. Schneidman-Duhovny, D., Hammel, M. & Sali, A. FoXS: a web server for rapid computation and fitting of SAXS profiles. *Nucleic Acids Res.* **38**, W540-W544 (2010).
